# Sulfur species and gold transport in arc magmatic fluids

**DOI:** 10.1038/s41561-024-01601-3

**Published:** 2024-12-16

**Authors:** Stefan Farsang, Zoltán Zajacz

**Affiliations:** Department of Earth Sciences, University of Geneva, Geneva, Switzerland

**Keywords:** Geochemistry, Volcanology

## Abstract

The sulfur species present in magmatic fluids affect the global redox cycle, the Earth’s climate and the formation of some of the largest and most economic ore deposits of critical metals. However, the speciation of sulfur under conditions that are relevant for upper crustal magma reservoirs is unclear. Here we combine a prototype pressure vessel apparatus and Raman spectroscopy to determine sulfur speciation in arc magmatic fluid analogues in situ over a range of geologically relevant pressure–temperature–redox conditions. We find that HS^−^, H_2_S and SO_2_ are the main sulfur species in the experimental fluids, while the concentrations of S^6+^ species and S_2_^−^ and S_3_^−^ sulfur radical ions are negligible, in contrast to previous experimental work. The measured gold solubilities in the experimental fluids are highest when sulfur is dominantly present as HS^−^ and H_2_S species and greatly exceed thermodynamic predictions. Our results imply that HS^−^, rather than sulfur radicals, accounts for the high solubilities of gold in magmatic–hydrothermal fluids. We also find that magmatic sulfur degassing is a redox process under oxidizing conditions and may lead to additional magma oxidation beyond that imparted by slab-derived fluxes and crystallization.

## Abstract (web summary)

There are three dominant sulfur species present in arc magmatic fluids: HS^−^, H_2_S and SO_2_. HS^−^ controls the mobilization and transport of gold in arc magmatic–hydrothermal systems, according to in situ experiments on arc magmatic fluid analogues.

## Main

Sulfur is an essential building block of proteins, and hence all life forms depend on its availability in the Earth’s surface environment^1^. The cycling of sulfur between surface and deep Earth reservoirs takes place predominantly through subduction and associated arc volcanism^2^. Ascending arc magmas, in which sulfur may exist in a range of valence states from 2− to 6+, release low-density magmatic fluids with a considerable part of their sulfur inventory partitioning into these fluids^2^. Sulfur in magmatic fluids that degas as volcanic gases is responsible for some of the most severe perturbations to the Earth’s climate following catastrophic volcanic explosions^3–7^, and changes in sulfur flux and speciation in volcanic gases have been suggested to trigger the Great Oxidation Event approximately 2.4 billion years ago^8–10^. Owing to the complex-forming capacity of sulfur species with certain metals, including gold, sulfur in magmatic fluids also controls metal solubility, transport and magmatic–hydrothermal (for example, porphyry Cu–Mo–Au) ore deposit formation^11–13^. Despite the tremendous importance of sulfur speciation for geologic, atmospheric and biogeochemical cycles, the experimental challenges associated with controlling the redox conditions corresponding to the sulfur redox-state transition at moderate pressures and high temperatures and the in situ monitoring of sulfur speciation have prohibited its direct experimental study under moderate-pressure and high-temperature conditions that are characteristic of upper crustal magma reservoirs in arc settings. Some of the first in situ sulfur speciation studies were conducted at moderate temperatures^14,15^, and whereas some experiments closely approached the relevant pressure–temperature conditions^16–19^, the oxygen fugacity ($f_{{\mathrm{O}}_2}$) could not be controlled directly in these.

Extensive volcanic gas sampling has shown that the predominant sulfur species in volcanic gases are hydrogen sulfide (H_2_S) and sulfur dioxide (SO_2_)^20,21^. These findings are in sharp contrast to experimentally constrained speciation models in magmatic fluids. For instance, under intermediate redox conditions, the trisulfur radical ion S_3_^−^ was interpreted as being the dominant sulfur-bearing aqueous species in geologic fluids above 250 °C and 0.5 GPa (ref. ^14^) and the one that assists the formation of world-class hydrothermal gold and platinum deposits^13,15,22,23^. Under oxidizing conditions, S^6+^ species were suggested to dominate, the volcanic emission of which was proposed to provide sulfate aerosol directly to the stratosphere, bypassing the need for the photochemical oxidation of magmatic SO_2_ (ref. ^24^). Such contrasting speciation models may arise due to experimental limitations, which include the lack of flexible, precise and accurate control over the redox conditions and the strong Raman resonance effecting the spectral bands of radical ion species S_2_^−^ and S_3_^−^ when the wavelength of excitation used lies inside the absorbance band of the respective species^25^. Although the use of lasers with such wavelengths enables the detection of extremely small amounts of chromophore species (for example, S_2_^−^ and S_3_^−^), it hinders their accurate quantification (Supplementary Table 1)^26,27^.

**Extended Data Fig. 1:**
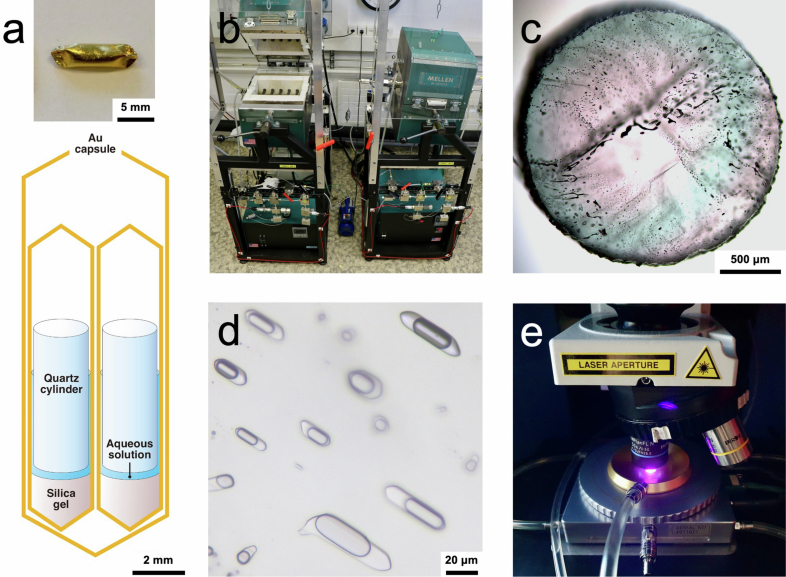
Our experimental setup and run products. (**a**) loaded inner Au capsule and the schematic drawing of an outer Au capsule holder accommodating two inner capsules; (**b**) prototype MHC pressure vessel setups; (**c**) polished quartz disk with SFI trapped at 2 kbar, 875 °C, and NNO-0.9; (**d**) a photomicrograph of some of these SFI; (**e**) Raman analysis of SFI conducted with the 405 nm excitation in a heating stage.

**Extended Data Fig. 2:**
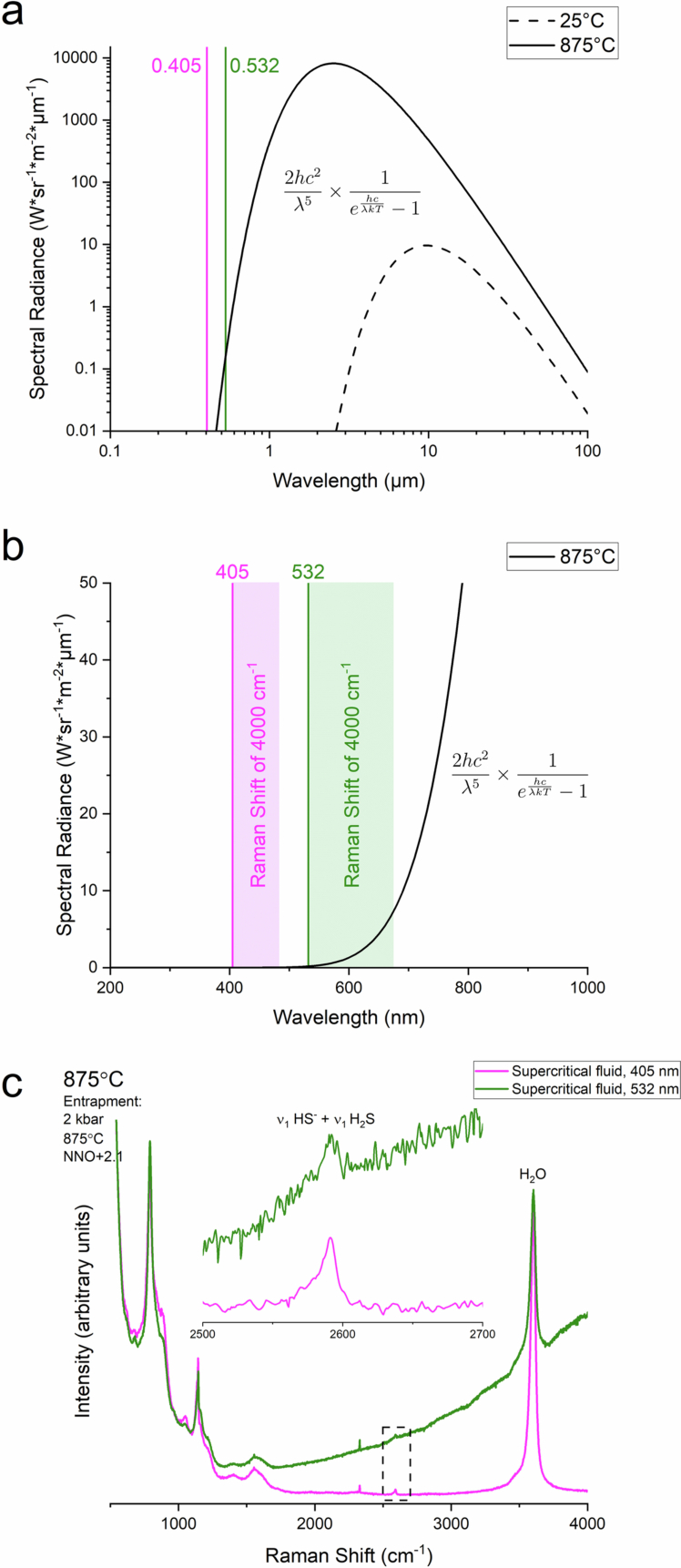
Thermal incandescence and its effect on Raman spectra. (**a**,**b**) Black-body spectra at room temperature (25 °C) and maximum experimental temperature (875 °C). Note that the broad thermal incandescence at 875 °C reaches the 532 nm line. Consequently, raw Raman spectra collected at 875 °C with the 532 nm excitation show an elevated background (**c**). Source data

**Extended Data Fig. 3:**
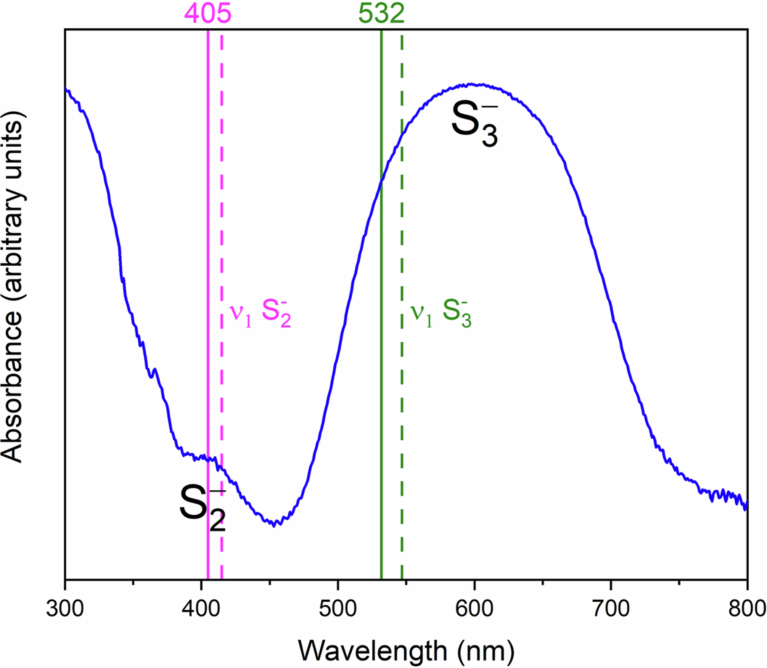
Diffuse reflectance spectrum of lazurite showing absorption bands of S_2_^–^ and S_3_^–^ radicals. The figure is modified after Farsang et al.^27^. The lazurite specimen is from Brazil, it is deposited at the Natural History Museum of Geneva and has a catalogue number 425.086. The 405 and 532 nm excitation lines used in this study (solid lines) and the corresponding ν_1_ S_2_^–^ and ν_1_ S_3_^–^ bands (dashed lines) are also shown. Due to the Raman resonance effect, the Raman signal of S_2_^–^ is strongly enhanced in its resonant spectrum collected with a 405 excitation and the signal of S_3_^–^ is strongly enhanced in its resonant spectrum collected with a 532 nm excitation. Therefore, the quantification of these species should only be attempted based on their non-resonant spectra, for example a spectrum collected with a 405 nm excitation will be non-resonant for S_3_^–^ and a spectrum collected with a 532 nm excitation will be non-resonant for S_2_^–^. Source data

In this study, we used a prototype, rapid-quench, externally heated molybdenum-hafnium carbide (MHC) pressure vessel assembly to trap sulfur-bearing supercritical fluids under controlled redox conditions within the pressure–temperature range of typical upper crustal magma reservoirs (Extended Data Fig. 1)^28^. Our experimental fluids are analogues of magmatic fluids associated with calc-alkaline magmatism in arc settings, which, following cooling, lead to the generation of porphyry deposits (Supplementary Information). The MHC vessel is equipped with a semipermeable hydrogen membrane^29^ that enables flexible, precise and accurate redox control and the entrapment of fluids in the form of synthetic fluid inclusions (SFIs) following the attainment of redox equilibrium. To analyse the trapped fluids, we re-heated the inclusions and used a combination of Raman laser wavelengths of 405 and 532 nm (ref. ^27^). Using the unique 405 nm excitation enabled the collection of high-temperature Raman spectra that are unaffected by thermal incandescence and thus have increased signal-to-noise ratios (Extended Data Fig. 2). Furthermore, the wavelength of 405 nm lies outside the absorbance band of the S_3_^−^ radical species, and the wavelength of 532 nm lies outside the absorbance band of the S_2_^−^ radical species, enabling the collection of Raman spectra that are unaffected by the signal enhancement of these species due to the Raman resonance effect (Extended Data Fig. 3)^25,27,30^. Using these experimental innovations, we determined the in situ speciation of sulfur in magmatic fluid analogues over the characteristic $f_{{\mathrm{O}}_2}$ range of arc magmas.

## Sulfur speciation in arc magmatic fluids

**Fig. 1:**
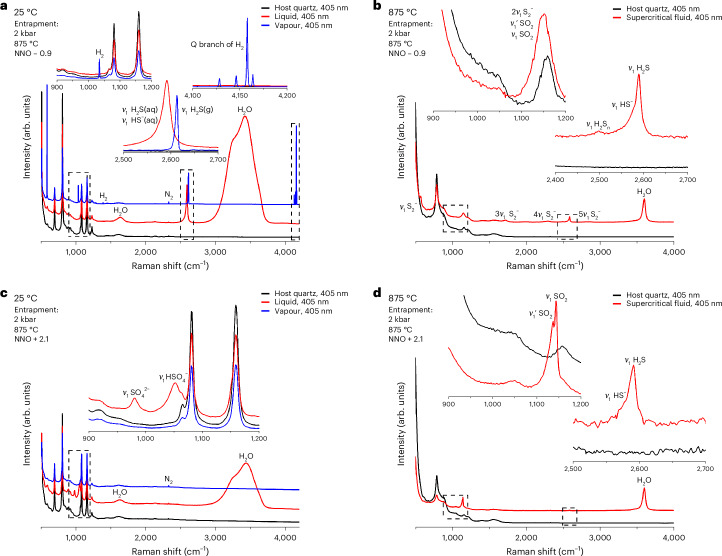
Contrasting sulfur speciation at ambient and entrapment temperatures under reducing and oxidizing conditions. **a**–**d**, Raman spectra of fluids trapped as ~20-µm-diameter SFIs under reducing (**a**,**b**) and oxidizing (**c**,**d**) conditions. The raw Raman spectra presented here are vertically offset for better readability, except for those in the insets. Insets show detailed spectral features in areas marked with dashed rectangles. Source data

**Extended Data Fig. 4:**
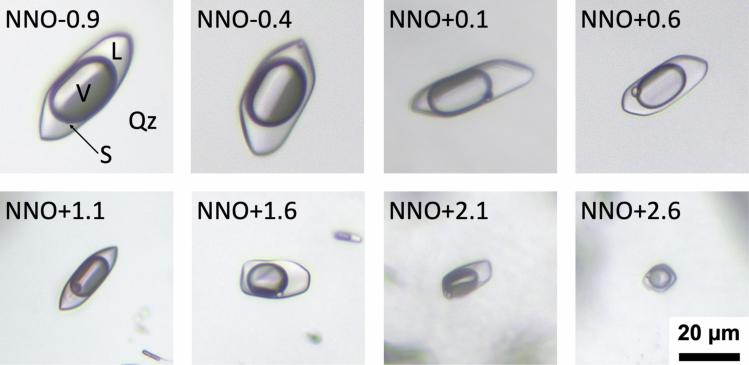
Plane polarized transmitted light microscope images (photomicrographs) of SFI trapped in quartz (Qz) host. At run conditions (2 kbar, 875 °C), a supercritical single-phase fluid is trapped that enters the vapour-liquid immiscibility field upon quenching. Consequently, a liquid phase (L) condenses, and a vapour bubble (V) nucleates. The indicated *f*O_2_ values refer to the entrapment conditions of SFI at 2 kbar and 875 °C. Note that the relative size of sulphur crystal (S) increases with increasing *f*O_2_ from almost invisible at the most reducing conditions. Furthermore, the size of SFI decreases with increasing *f*O_2_.

Sulfur speciation was studied by in situ Raman spectroscopy on fluids trapped as ~20-µm-diameter SFIs at 200 MPa (2 kbar), 875 °C and under redox conditions that are representative of arc-related magmatic systems, ranging from NNO − 0.9 to NNO + 2.6 (where ΔNNO is the deviation of $\log f_{{\mathrm{O}}_2}$ from the Ni–NiO buffer). In addition, a fluid was sampled at NNO + 7.4. The speciation in quenched fluids at room temperature (25 °C) is in sharp contrast to that observed under their entrapment pressure, temperature and $f_{{\mathrm{O}}_2}$ conditions (Table 1). At 25 °C, all SFIs contain a solid sulfur crystal, a liquid and a vapour phase (Extended Data Fig. 4). In the liquid, aqueous sulfide species H_2_S and HS^−^ dominate the reducing end of the experimental $f_{{\mathrm{O}}_2}$ conditions (Fig. 1a), whereas sulfate species HSO_4_^−^ and SO_4_^2−^ are the most abundant under oxidizing conditions (Fig. 1b). Close to the sulfide–sulfate transition, SO_4_^2^^−^ prevails over HSO_4_^−^, and moving to higher $f_{{\mathrm{O}}_2}$ results in the increase of the HSO_4_^−^/SO_4_^2−^ ratio. In the vapour bubble, H_2_S and H_2_ gas is present under reducing conditions (Fig. 1a), and no sulfur species were detected under oxidizing conditions.

**Table 1:** Sulfur species and H_2_ gas detected in SFIs

$f_{{\mathrm{O}}_2}$ (ΔNNO)	Liquid phase, 25 °C	Vapour phase, 25 °C	Supercritical fluid phase, 875 °C
Starting fluid composition: H_2_O + 1 mol NaCl per kg H_2_O + 5 mol% H_2_SO_4_			
7.4	HSO_4_^−^, SO_4_^2−^		SO_2_, SO_4_^2−^
2.6	HSO_4_^−^, SO_4_^2−^		SO_2_
2.1	HSO_4_^−^, SO_4_^2−^		SO_2_, HS^−^, H_2_S
1.6	HSO_4_^−^, SO_4_^2−^		SO_2_, HS^−^, H_2_S, S_2_^−^
1.1	HSO_4_^−^, SO_4_^2−^	H_2_S	SO_2_, HS^−^, H_2_S, S_2_^−^
0.6	HSO_4_^−^, SO_4_^2−^		HS^−^, H_2_S, SO_2_, S_2_^−^
0.1	H_2_S, HS^−^, SO_4_^2−^	H_2_S, H_2_	HS^−^, H_2_S, SO_2_, S_2_, S_3_^−^
−0.4	H_2_S, HS^−^, SO_4_^2−^	H_2_S, H_2_	HS^−^, H_2_S, H_2_S_*n*_, SO_2_, S_2_^−^, S_3_^−^
−0.9	H_2_S, HS^−^	H_2_S, H_2_	HS^−^, H_2_S, H_2_S_*n*_, SO_2_, S_2_^−^, S_3_^−^

Note that all SFIs contained a solid sulfur crystal at 25 °C (Extended Data Fig. 4). The indicated $f_{{\mathrm{O}}_2}$ values refer to the entrapment conditions of the SFIs at 2 kbar and 875 °C.

**Fig. 2:**
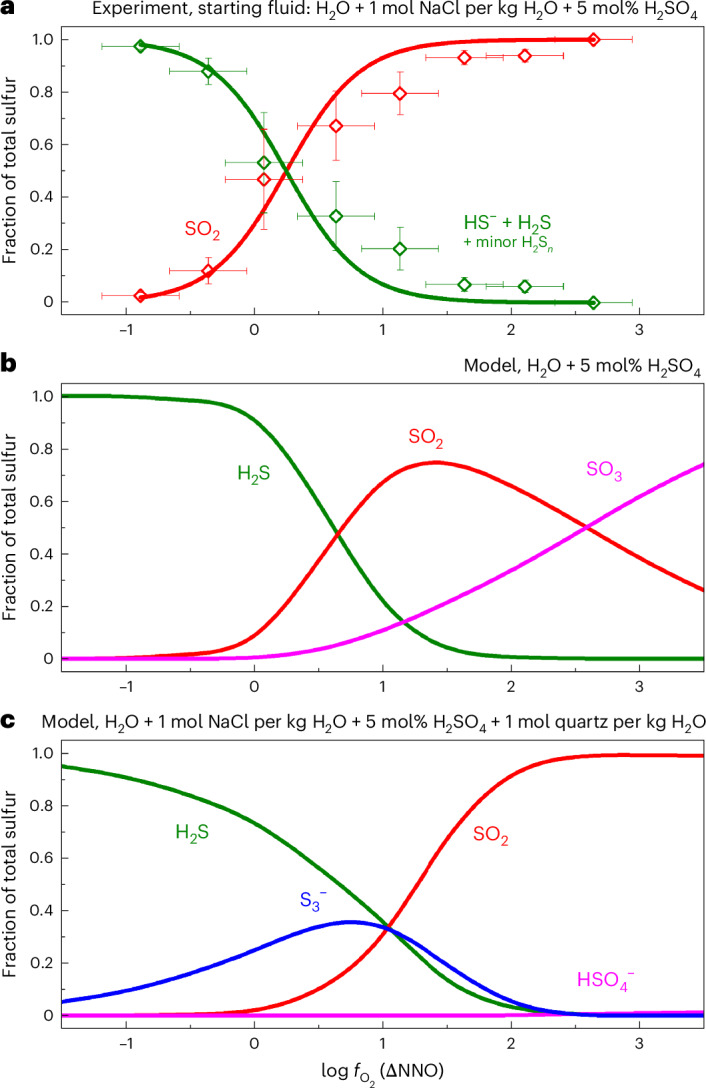
Sulfur speciation in an aqueous supercritical fluid phase at 2 kbar and 875 °C. **a**–**c**, Sulfur speciation as determined experimentally in this study (**a**), calculated using the experimentally derived equilibrium constants of Binder and Keppler^7^ (**b**) and calculated using the thermochemical data reported in Supplementary Table 5 with the S_3_^−^ radical included in the calculation (**c**). Note that only those sulfur species that reached a concentration of at least 1% of total sulfur at any of the considered $f_{{\mathrm{O}}_2}$ values are visualized. In **a**, the horizontal error bars correspond to the estimated 2*σ* error in $f_{{\mathrm{O}}_2}$ (that is, $0.3 \log f_{{\mathrm{O}}_2}$ units^28^) and the vertical error bars correspond to the estimated 1*σ* error on sulfur speciation that arises from the peak fitting of one Raman spectrum per experiment. Source data

Upon heating, sulfate protonation takes place, followed by the melting of the sulfur crystal, sulfur radical formation and sulfur comproportionation reactions in which two species with sulfur in different oxidation states form a species with sulfur in an intermediate oxidation state (Supplementary Information and Supplementary Fig. 1). First, sulfur and HSO_4_^−^ (bisulfate), then H_2_S and bisulfate react to form SO_2_. At the entrapment temperature of 875 °C, sulfide species (HS^−^ and H_2_S) and SO_2_ are the dominant sulfur species in the supercritical fluid phase (Fig. 1c,d) with the sulfide–SO_2_ equimolality situated at NNO + 0.2 (Fig. 2). The equilibrium between H_2_S and SO_2_ can be described according to Binder and Keppler^7^:

1 $$2{{\mathrm{H}}}_{2}{\mathrm{S}}({{\mathrm{aq}}})+3{{\mathrm{O}}}_{2}({{\mathrm{aq}}})=2{\mathrm{S}}{{\mathrm{O}}}_{2}({{\mathrm{aq}}})+2{{\mathrm{H}}}_{2}{\mathrm{O}}$$

with an equilibrium constant, *K*_1_, of

2 $${K}_{1}=\frac{{f_{{\mathrm{S}}{\mathrm{O}}_2}}^{2}\times {f_{{{\mathrm{H}}}_2{{\mathrm{O}}}}}^{2}}{{f_{{{\mathrm{H}}}_2{{\mathrm{S}}}}}^{2}\times {f_{{{\mathrm{O}}}_2}}^{3}},$$

where $f_{{\mathrm{S}}{\mathrm{O}}_2}$, $f_{{\mathrm{H}}_2{\mathrm{O}}}$ and $f_{{\mathrm{H}}_2{\mathrm{S}}}$ are the fugacities of SO_2_, H_2_O and H_2_S, respectively.

Fitting our experimental data (Fig. 2a) yields log *K*_1_ = 42.5. The concentration of HS^−^ is approximately twice the concentration of H_2_S over the $f_{{\mathrm{O}}_2}$ range of their occurrence (Supplementary Table 2). This finding is in sharp contrast to thermodynamic models that predict negligible amounts of HS^–^ in arc magmatic fluids (Fig. 2b,c and Supplementary Table 2). The secondary equilibrium between H_2_S and HS^−^ can be described by:

3 $${{\mathrm{H}}}_{2}{\mathrm{S}}({{\mathrm{aq}}})={\mathrm{H}}{{\mathrm{S}}}^{-}({{\mathrm{aq}}})+{{\mathrm{H}}}^{+}({{\mathrm{aq}}}).$$

At least some of the HS^−^ may be stabilized by complexing with the abundant Na^+^ cations in our supercritical fluids.

**Fig. 3:**
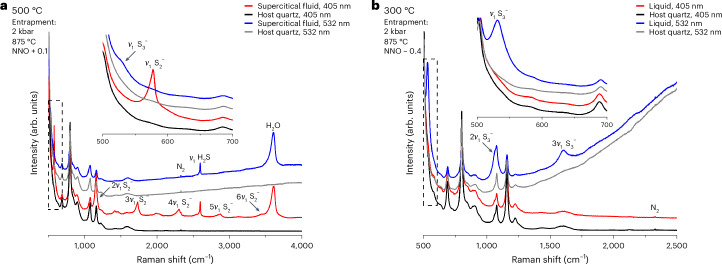
Raman spectra showing the contrasting detection of S_2_^−^ and S_3_^−^ with different excitation wavelengths. **a**,**b**, Spectra of S_2_^−^ (**a**) and S_3_^−^ (**b**) were collected under temperature and $f_{{\mathrm{O}}_2}$ conditions at which the respective species showed the strongest signals in the resonant Raman spectra of fluids containing 1 mol NaCl per kg H_2_O. S_2_^−^ remained below the detection limit in its non-resonant Raman spectrum collected at 532 nm excitation (**a**), and S_3_^−^ remained below the detection limit in its non-resonant Raman spectrum collected at 405 nm excitation (**b**). The raw Raman spectra presented here were vertically offset for better readability. Insets show detailed spectral features in areas marked with dashed rectangles. Source data

Minor sulfur species detected in the supercritical fluid at 875 °C include sulfur radical ions, polysulfanes (H_2_S_*n*_; *n* > 1) and bisulfate. Around 200 °C, S_2_^−^ and S_3_^−^ radical ions appear in the liquid and remain stable up to the entrapment temperature of 875 °C under a wide range of $f_{{\mathrm{O}}_2}$ conditions. The S_2_^−^ and S_3_^−^ radical ions are chromophore species^30,31^ and are only detected owing to the resonance Raman effect (Extended Data Fig. 3). In their respective resonant Raman spectra, S_2_^−^ and S_3_^−^ show their maximum intensities around 500 and 300 °C, respectively (Fig. 3). In their respective non-resonant Raman spectra, their concentration remains below the detection limit under all investigated conditions (Fig. 3). These observations suggest that previous attempts at quantifying sulfur radical species—all of which used excitation wavelengths that were inside the absorbance bands of the respective sulfur radical species (Supplementary Table 1)—severely overestimated their concentration and geological importance. Nevertheless, the occurrence and formation of these exotic species is very interesting (Supplementary Information and Supplementary Figs. 2–5). Polysulfanes, with a general formula of H_2_S_*n*_ (*n* > 1)^32,33^, were observed under the two most reducing sets of conditions (Supplementary Table 2). Polysulfanes have recently been described from both natural^34,35^ and synthetic^19^ fluid inclusions, in which they also coexist with H_2_S. Given the chemical similarity of polysulfanes to H_2_S (ref. ^19^), we grouped them together with S^2−^ species in the speciation diagram (Fig. 2). Finally, minor bisulfate remained stable up to 700 °C in our fluids, and sulfate occurred upon condensation of a brine following SFI oxidation at high temperatures (Supplementary Information and Supplementary Figs. 6–10).

**Fig. 4:**
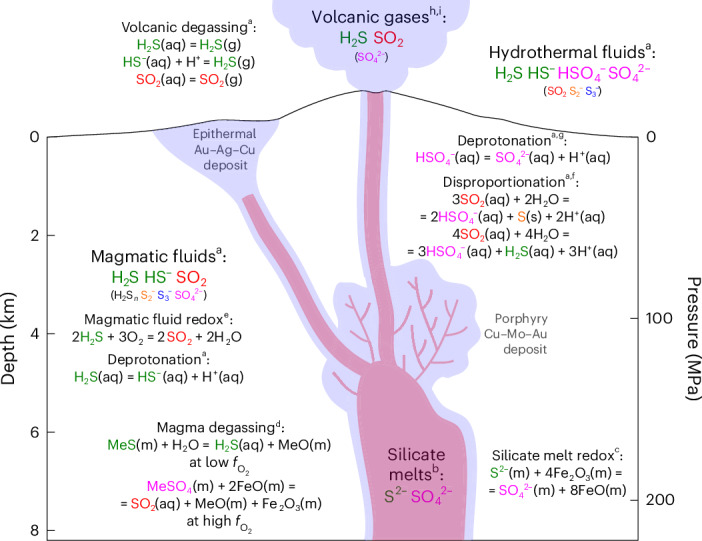
Sulfur speciation and equilibria in a calc-alkaline magmatic system related to arcs. ^a^This work. ^b^Jugo et al.^38^. ^c^Bénard et al.^41^. ^d^Ding et al.^51^. ^e^Binder and Keppler^7^. ^f^Drummond^43^. ^g^Ni and Keppler^18^. ^h^Wallace and Edmonds^20^. ^i^Roberts et al.^47^. (aq), aqueous species; (g), gaseous species; (m), species in silicate melt; (s), solid species; Me, divalent metal (for example, Ca^2+^).

In silicate melts, sulfide and sulfate are the only major sulfur species with a sharp transition between the predominance of the 2− and 6+ oxidation states with increasing $f_{{\mathrm{O}}_2}$ (refs. ^36–39^). The lack of S^6+^ species in arc magmatic fluids implies that magmatic degassing is indeed a redox process under oxidizing conditions. Sulfur is reduced from S^6+^ to S^4+^, and iron, being the most abundant redox-sensitive element in the silicate melt, is probably oxidized from Fe^2+^ to Fe^3+^ upon SO_2_ degassing (Fig. 4):

4 $${{\mathrm{MeS}}}{{\mathrm{O}}}_{4}({\mathrm{m}})+2{{\mathrm{FeO}}}({\mathrm{m}})={\mathrm{S}}{{\mathrm{O}}}_{2}({{\mathrm{aq}}})+{{\mathrm{MeO}}}({\mathrm{m}})+{{{\mathrm{Fe}}}}_{2}{{\mathrm{O}}}_{3}({\mathrm{m}}),$$

where Me is a divalent metal. Consequently, sulfur degassing may help to further oxidize arc magmas after initial oxidation by subducted slab-derived fluxes^40,41^ or crystallization of silicate minerals that incorporate iron dominantly as Fe^2+^ (ref. ^42^).

## Sulfur species in other geologic fluids and volcanic gases

We showed that sulfur speciation in arc magmatic fluids is unquenchable because several reactions are taking place during cooling from magmatic temperatures: SO_2_ undergoes disproportionation, first to H_2_S and bisulfate and then to sulfur and bisulfate below ~500 °C (ref. ^43^); sulfur radical species S_2_^−^ and S_3_^−^ become unstable below ~200 °C (ref. ^14^); and HSO_4_^–^ may undergo deprotonation below 100 °C (ref. ^18^) (Fig. 4). One of the unique features of shallow (<10 km), high-temperature (>600 °C) arc magmatic fluids is their low density (typically <0.5 g cm^−3^) that stabilizes SO_2_ over sulfate species. Below ~500 °C, SO_2_ destabilizes and sulfate species HSO_4_^−^ and SO_4_^2−^ become the dominant oxidized sulfur species in the higher-density moderate-temperature hydrothermal fluids. It is therefore likely that in high-density geologic fluids, including magmatic brines, hydrothermal fluids and subduction-zone fluids, sulfate species instead of SO_2_ dominate under oxidizing conditions.

In the studied supercritical fluids, sulfide species HS^−^ and H_2_S dominate the reducing end and SO_2_ dominates the oxidizing end of our experimental spectrum. However, H_2_S and SO_2_ are the only widely observed sulfur species outgassing at volcanic vents^20,21^. HS^−^ is absent because it is unstable in the low-density volcanic vapour phase and probably reacts with H^+^ to form H_2_S upon volcanic degassing. In the studied arc magmatic fluid analogues, we detected no sulfuric acid (H_2_SO_4_). In previous studies, S^6+^ species were suggested to dominate arc magmatic fluids at the high end of the $f_{{\mathrm{O}}_2}$ range of arc magmatism, either because speciation was determined on the basis of quenched fluids^7^ or because of the erroneous assignment of the ~1,137 cm^−1^ band to H_2_SO_4_ (ref. ^18^) (see discussion in Supplementary Information and Supplementary Figs. 11 and 12). It should be noted that sulfate aerosol or sulfuric acid have been detected at some volcanic vents, including that of the passively degassing Masaya Volcano, Nicaragua^44,45^ and the Bezymianny Volcano, Kamchatka^46^. Although the concentration of sulfate in volcanic gases is generally very small, with typical SO_4_^2−^/SO_2_ molar ratios ranging from 0.00002 to 0.01 (ref. ^47^), its presence was used to justify sulfur speciation models for magmatic fluids which proposed SO_3_ (sulfur trioxide) dominance under oxidizing conditions^7,18^. Sulfate was suggested to be a primary magmatic vapour-phase constituent^44^, formed via the oxidation of SO_2_ to sulfate precursor SO_3_ in the magmatic vapour^44^, and in volcanic domes, via the oxidation of SO_2_ due to circulation of air^46^. At 875 °C we detected minor S^6+^ species only in the supercritical fluid trapped at $f_{{\mathrm{O}}_2}$ = NNO + 7.4, which is not representative of arc magmatic fluids. Besides the scenarios outlined in ref. ^44^, which require highly oxidizing magmatic fluids, we suggest two alternative explanations for sulfate detection. After switching off the heating stage in our experiments, a sulfur crystal precipitated within seconds during cooling, showing that the disproportionation of SO_2_ to sulfur and sulfate (SO_4_^2−^ or HSO_4_^−^) is a very rapid process. The apparent emission of sulfate and the simultaneous precipitation of elemental sulfur may therefore take place due to sulfur disproportionation at sites of passive degassing where relatively slow degassing rates are combined with a large temperature drop. We also showed that the oxidation of SFIs and a density drop due to diffusive water loss from the SFI at high temperatures may lead to the condensation of a high-density alkali- and sulfate-rich brine (see discussion in Supplementary Information). In nature, such brines could theoretically form due to the decompression of supercritical fluids in the volcanic conduit before eruption. Iron in the magma acting as an oxidizing agent for sulfur partitioning into the brine or hydrogen loss to the vapour may account for redox balance. Sulfate could therefore be detected during the sudden release of such brines upon volcanic eruptions. Our experimental sulfur speciation suggests that the large amounts of stratospheric sulfuric acid aerosols indeed originate from the photochemical oxidation of SO_2_ (ref. ^24^).

Given the dependence of sulfur speciation on $f_{{\mathrm{O}}_2}$ (equation (1)), existing data on the molar SO_2_/H_2_S ratios of natural volcanic gases^48^ may be used as a proxy for $f_{{\mathrm{O}}_2}$ conditions after correcting for the effect of the decompression induced $f_{{\mathrm{H}}_2{\mathrm{O}}}$ drop.

## Transport of metals in magmatic–hydrothermal systems

Our observation that the concentration of sulfur radical ions in arc magmatic fluid analogues is very low (that is, it falls below the detection limit of respective non-resonant Raman spectra under all experimental conditions) is in sharp contrast to previous studies that suggested S_3_^−^ to be the dominant stable form of sulfur in aqueous solutions above 250 °C and 0.5 GPa (ref. ^14^). Even at magmatic temperatures, thermodynamic models that include thermochemical data for S_3_^−^ (ref. ^15^) predict very high S_3_^−^ concentrations that account for more than 30% of total sulfur under intermediate redox conditions (Fig. 2c). However, the excitation wavelengths used in these studies (632, 473 and 532 nm) all lie inside the absorption band of S_3_^−^ (Extended Data Fig. 2), leading to a Raman resonance effect and, consequently, a strong enhancement of its Raman signal. If S_3_^−^ is present in any appreciable amount, it should be easily visible even in non-resonant (for example, 405 nm) spectra, as demonstrated in the case of lazurite^27^. At lower temperatures that are characteristic of hydrothermal systems, sulfide precipitation and consequent drop in sulfur concentration and redox changes in the fluid may further reduce the stability of sulfur radical species.

**Fig. 5:**
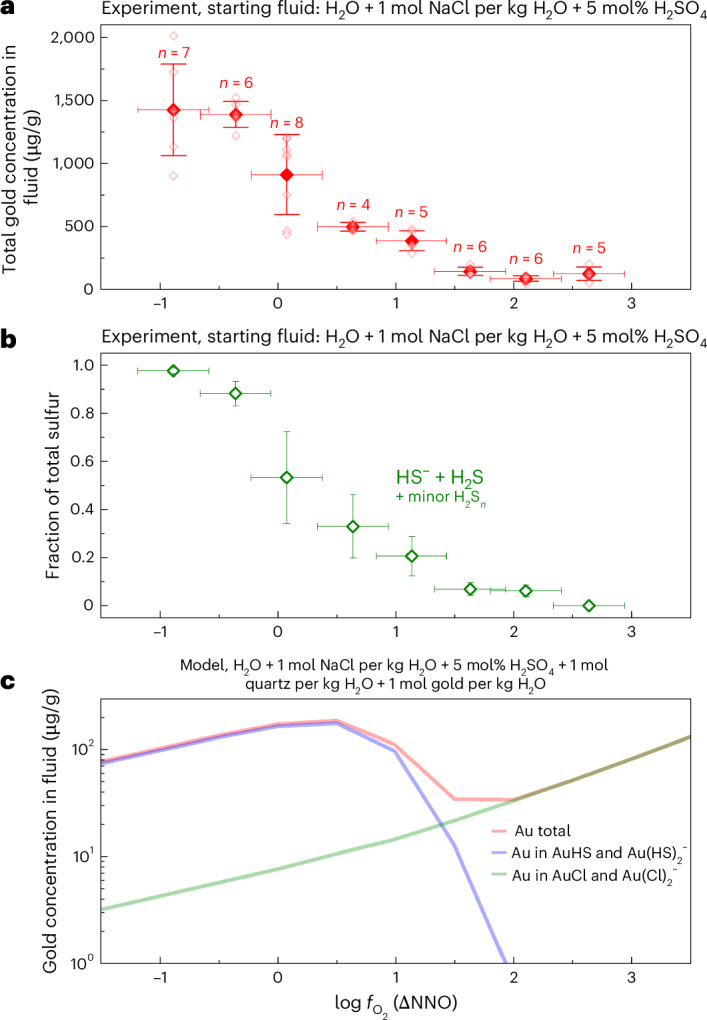
Gold solubility at 2 kbar and 875 °C. **a**,**b**, Total gold solubility (**a**) and the corresponding fraction of reduced sulfur species (**b**) in our experimental fluids. In **a**, the drop in gold solubility with increasing $f_{{\mathrm{O}}_2}$ corresponds to the drop in HS^−^ + H_2_S in the fluid. No maximum in gold solubility is observed at the centre of the sulfur redox transition (~NNO + 0.2) where the concentration of sulfur radical ions should be the highest (Fig. 2c). In **a** and **b**, the horizontal error bars correspond to the estimated 2*σ* error in $f_{{\mathrm{O}}_2}$ (that is, $0.3 \log f_{{\mathrm{O}}_2}$ units^28^); in **a**, the vertical error bars correspond to the estimated 1*σ* error around the arithmetic mean of the data shown as filled symbols, and in **b** they correspond to the estimated 1*σ* error on sulfur speciation that arises from the peak fitting of one Raman spectrum per experiment. **c**, Gold solubility and speciation predicted by thermodynamic model calculations. Note that the model severely underestimates the gold solubility and the concentration of HS^−^ and Au–HS^–^ species in the fluid. Moreover, the Au–HS^–^ species under reducing conditions are dominated by Au(HS)_2_^−^, and Au–OH complexes are not shown as their predicted concentration remained below 1 µg g^−1^ under all $f_{{\mathrm{O}}_2}$ conditions. Note that the sulfur redox transition occurs at about $0.5 \log f_{{\mathrm{O}}_2}$ units higher in the model calculation (Fig. 2), and the predicted gold solubility pattern is offset accordingly compared with the experimental data. Source data

Our experiments also show that, in arc magmatic fluids, HS^−^ is the most abundant reduced sulfur species. To test whether this HS^−^ can account for high metal solubilities in natural arc magmatic fluids, we used laser ablation inductively coupled plasma mass spectrometry (LA-ICP-MS) to measure the gold concentrations of our experimental fluids that were in equilibrium with gold (the capsule material). The analysis of our experimental fluids shows that gold solubility increases sharply with decreasing $f_{{\mathrm{O}}_2}$, closely mirroring the increase in concentration of HS^−^ in the fluid, suggesting gold is transported predominantly as Au–HS^−^ species (Au(HS)_2_^−^ and AuHS). Gold solubility is 129 ± 52 µg g^−^^1^ in the most oxidizing arc magmatic fluid analogue, whereas it reaches a remarkable 1,420 ± 360 µg g^−1^ (0.14 wt%) in the most reducing fluid (Fig. 5 and Supplementary Table 4). A relatively reducing arc magmatic fluid can therefore transport an order of magnitude more gold than a relatively oxidizing fluid. If the thermochemical data for S_3_^−^ of Pokrovski et al.^13^ were correct, we should see abundant S_3_^−^ under $f_{{\mathrm{O}}_2}$ conditions corresponding to the sulfide–SO_2_ transition (Fig. 2 and Supplementary Fig. 4), accompanied by a peak in gold solubility. However, we do not see either of these. Instead, our data show that HS^−^ can effectively mobilize and transport very high concentrations of gold in the absence of abundant sulfur radical ions in the fluid. Moreover, the experiments of Hu et al.^19^ indicate that, given the chemical similarities of polysulfanes (H_2_S_*n*_) to H_2_S, their deprotonation products HS_*n*_^−^ and S_*n*_^2−^ may also participate in the transport of gold. Finally, at oxidizing conditions, some gold likely complexes with chlorine and gets transported as Au–Cl complexes, as suggested by thermodynamic calculations (Fig. 5c).

The severe underestimation of the concentration of HS^−^ in arc magmatic fluids by the thermodynamic model calculations (Supplementary Table 2) point to incorrect thermochemical data for aqueous HS^−^. Given that the concentration of sulfur radical species remained below the detection limit of their respective non-resonant Raman spectra even at moderate temperatures, at which their resonant Raman signals are the strongest (Fig. 3), it is likely that HS^−^ dominates gold mobilization and transport in (lower-temperature) hydrothermal fluids too, as suggested by the recent experimental studies of Trigub et al.^49^ and Tagirov and colleagues^50^. Finally, HS^−^ probably accounts for the high solubilities and transport of certain other chalcophile metals such as platinum in magmatic–hydrothermal fluids, the transport of which has previously been suggested to be controlled by S_3_^−^ radical ions^23^.

## Methods

### Experimental strategy

First, sulfur-bearing fluids were sampled under run pressure–temperature conditions using a modified version of the SFI technique^52^. The experiments were conducted at 2,000 ± 20 bar (200 ± 2 MPa) and 875 ± 10 °C, and at eight oxygen fugacity ($f_{{\mathrm{O}}_2}$) values ranging from 0.9 log units below that of the Ni–NiO buffer (NNO − 0.9) to 2.6 log units above that of the Ni–NiO buffer (NNO + 2.6) (Table 1). In addition, a fluid was also sampled at NNO + 7.4. The chosen $f_{{\mathrm{O}}_2}$ values covered the $f_{{\mathrm{O}}_2}$ range that is characteristic of most natural arc magmas^53,54^ and the S^2−^ to S^6+^ (sulfide to sulfate) transition in silicate melts under the run conditions^55^. To represent the composition of a typical arc magmatic fluid exsolving from the silicate melt in the upper crust, a vapour-like low-salinity model aqueous fluid (1 mol NaCl per kg H_2_O and 5 mol% (~7 wt%) sulfur) was chosen for our experiments^55–57^. To investigate the effect of fluid salinity on the formation of sulfur radicals, four additional capsules were prepared, each with a fluid consisting of 5 mol% sulfur but different salinities (Supplementary Table 3). The synthesized fluid inclusions were subsequently re-heated to their entrapment temperature of 875 °C and the sulfur speciation inside them was determined by in situ Raman spectroscopy.

### Capsule preparation

The capsules containing the experimental-phase assemblage were made of gold tubing (>99.99% purity, Refining Systems). Each gold capsule (12 mm long, 3 mm outer diameter (OD), 2.7 mm inner diameter (ID)) was loaded with an inclusion-free cylindrical quartz chip (6 mm long, 2.1 mm diameter), dehydrated silica gel (~3 mg) to promote fracture healing and aqueous starting solution (~5 µl) (Extended Data Fig. 1a). The quartz cylinder was cut with its long axis perpendicular to the *c* crystallographic axis of quartz, which is the fast direction for hydrogen diffusion in the quartz structure^58,59^, to slow down hydrogen diffusion during the high-temperature Raman experiments by forcing hydrogen to follow a much longer diffusion path to the sidewalls of the disc. The solution consisted of 1 mol NaCl per kg H_2_O and 5 mol% sulfur added as H_2_SO_4_. Double-distilled and deionized water (with a resistivity of 18 MΩ cm), high-purity NaCl (>99.999%, Sigma Aldrich) and reagent-grade H_2_SO_4_ (96 wt%, Carlo Erba) were used for preparation of the solutions. The capsules were welded using a PUK U5 welder (Lampert) and checked for potential weight loss during welding by weighing them before and after welding. The integrity of the welds was checked by holding the capsules at 130 °C for at least 30 min and subsequent weighing. In each experiment, two capsules were placed into a holder (5 mm OD, 4.7 mm ID), also made of gold tubing. This outer capsule was welded at one end and just crimped at the other (as it was only used to ensure that the smaller capsules placed inside were held in place during the experiment). A 4-mm-long ceramic spacer was placed in between the membrane and the outer capsule to prevent alloying of the nickel–copper alloy with gold at high temperatures.

In the additional experiments that aimed to investigate the effect of the fluid salinity on the formation of sulfur radicals, the fluid in the four capsules consisted of 5 mol% sulfur added as H_2_SO_4_ and different amounts of salt dissolved in water. In addition to the solution of 1 mol NaCl per kg H_2_O prepared in the previous set, these fluids were NaCl/KCl-free or contained 1 mol KCl per kg H_2_O, 4 mol NaCl per kg H_2_O or 4 mol KCl per kg H_2_O.

For fluid entrapment at NNO + 7.4, a small capsule was placed into a larger capsule (20 mm long, 5 mm OD, 4.7 mm ID) made of gold tubing along with a 1:9 molar mixture (~100 mg) of Ru–RuO_2_ buffer and water (25 µl).

### Experimental technique

SFIs were made in a rapid-quench, externally heated MHC pressure-vessel assembly at the Department of Earth Sciences, University of Geneva (Extended Data Fig. 1b). This prototype apparatus enables flexible, precise and accurate redox control via a custom-designed semipermeable hydrogen membrane while maintaining a rapid-quench capability^28^.

During experimental runs, the MHC vessel was first flushed with argon (~100 bar; 99.999% purity) at least four times to remove all traces of air, then pressurized to ~800 bar. Subsequently, the vessel was heated to 875 ± 10 °C, resulting in a pressure increase to 2,000 ± 20 bar. The experimental pressure was monitored using a factory-calibrated digital pressure transducer and is constrained with better than 20 bar accuracy and precision. The experimental temperature was measured using a K-type thermocouple attached to the external surface of the Inconel sheath that protected the MHC vessel from oxidation. The internal temperature in the vessel was cross-calibrated against this external thermocouple beforehand. The temperature gradient over the capsule length (12 mm) was <10 °C, and the temperature was constrained with better than 10 °C accuracy.

Oxygen fugacity values ranging from NNO − 0.9 to NNO + 2.6 were imposed by controlling the hydrogen fugacity ($f_{{\mathrm{H}}_2}$) in the argon pressure medium using a metal membrane that is permeable to hydrogen (Shaw membrane^29^)^28^. Hydrogen was supplied to the Shaw membrane from a one-litre-volume reservoir to ensure constant $f_{{\mathrm{H}}_2}$. The pressure in the reservoir was monitored using a factory-calibrated digital pressure transducer. The H_2_ gas in turn diffused through the capsule walls and reacted with the water inside to impose $f_{{\mathrm{O}}_2}$ inside the capsule through the decomposition of water:

5 $${2{\mathrm{H}}}_{2}{\mathrm{O}}={2{\mathrm{H}}}_{2}+{{\mathrm{O}}}_{2}.$$

The equilibrium constant of the water decomposition reaction, *K*, was calculated using thermochemical data from the JANAF database^60^ to obtain the desired $f_{{\mathrm{H}}_2}$ inside the capsule:

6 $$f_{{\mathrm{H}}_2}=f_{{{\mathrm{H}}}_{2}{\mathrm{O}}}\sqrt{\frac{K}{f_{{\mathrm{O}}_2}}}.$$

Note that the $f_{{\mathrm{H}}_2}$ inside the vessel and thus the capsule is lower than that measured in the reservoir by the pressure transducer. The efficiency of the Shaw membrane was tested via (1) a two-membrane test in which one of the membranes was used to supply hydrogen and the other membrane was used to monitor it and (2) a bracketing test performed during experimental runs. We found that with the membrane we used throughout the experiments, 80% of the imposed hydrogen pressure is reached as equilibrium $f_{{\mathrm{H}}_2}$ and corrected the $f_{{\mathrm{H}}_2}$ values accordingly. The estimated 2*σ* error in $f_{{\mathrm{O}}_2}$ is $0.3 \log f_{{\mathrm{O}}_2}$ units^28^.

The vapour-like low-density supercritical aqueous fluids (~0.4 g cm^−3^) were trapped as SFIs in cylindrical quartz chips. The quartz was fractured in situ during the experiment after 72 h of equilibration at 875 ± 10 °C, by rotating the vessel–furnace assembly to drop the capsule to the water-cooled end and then back to the hot end of the vessel, similarly to the technique of Sterner and Bodnar^52^ and Li and Audétat^61^. The thermal shock and the rapid transition from β- to α-quartz generated a dense interconnected network of fractures, which was subsequently allowed to heal for 72 h, leading to the formation of SFIs. Despite the large number of fractures produced, the mechanical integrity of the chip was preserved. Before opening, the recovered capsules were checked once again for potential weight loss. The quartz cylinders retrieved from the capsules were subsequently cut into discs of about 1 mm thickness, embedded in Crystalbond 509 resin and polished to 1 μm fineness (Extended Data Fig. 1c). After polishing, the resin was completely removed via dissolution in acetone. SFIs suitable for the Raman analysis were selected using a petrographic microscope (Extended Data Fig. 1d).

The duration of the experiments was determined considering the time required for (1) the achievement of redox equilibrium between the capsule contents and the pressure medium before the in situ fracturing of the quartz and (2) the healing of the quartz fractures to form fluid inclusions after the fracturing. The time required to achieve redox equilibrium was estimated via model calculation. In the capsules, sulfur is the only constituent with a considerable redox capacity, and $f_{{\mathrm{H}}_2}$ is dependent on the H_2_S/SO_2_ ratio in the fluid. For each time step of our model, the H_2_S/SO_2_ ratio was calculated using the equilibrium constant of equation (1), as determined by Binder and Keppler^7^, and that of the water decomposition reaction (equation (5)). The calculated $f_{{\mathrm{H}}_2}$ was then dynamically linked to the rate of diffusion of H_2_ through the walls of the capsule using the equations of Chou^62^. Our calculations predicted that the $f_{{\mathrm{H}}_2}$ in the capsule achieved stable equilibrium with the $f_{{\mathrm{H}}_2}$ imposed in the vessel (defined as reaching 99.9% of the imposed $f_{{\mathrm{H}}_2}$ value) within 3 h after the start of the experiment at 875 °C. The time slot of three days that was provided for the experiments was therefore sufficient by a large safety margin to achieve redox equilibrium within the capsule before the in situ fracturing of the quartz. After the fracturing, three days were allowed for the healing of the quartz fractures. In previous experiments conducted at magmatic temperatures, similar timescales were applied to achieve redox equilibrium and post-entrapment healing^12,63–65^.

For fluid entrapment at NNO + 7.4 imposed by the Ru–RuO_2_ buffer, a traditional MHC pressure-vessel apparatus with no Shaw membrane was used.

### Selection of fluid inclusions for Raman analysis

An ideal fluid inclusion for Raman analysis has a high optical clarity, sufficient size and a shallow depth below the host surface^66^. Furthermore, the host–inclusion boundary in the laser path should be as perpendicular as possible to the incoming laser, because the difference in refractive index between the inclusion and its host mineral can cause the inclusion to act like a lens^66^. It is also important to ensure that the position of the laser focus remains the same throughout the heating experiment^67^.

We found that the most ideally suited SFIs are those around 20 µm long and situated around 100 µm below the surface of the quartz disc. Larger SFIs, or those sitting closer to the surface, generally decrepitated on heating. Before the collection of each Raman spectrum, scans along the *z* axis were performed to find the intensity maximum of the water stretching band. Raman spectra were then obtained at these depths.

### Raman spectroscopy

Raman spectra were collected using a confocal LabRAM HR Evolution (HORIBA Scientific) Raman spectrometer with an 800 mm focal length at the Department of Earth Sciences, University of Geneva. The spectrometer was equipped with a liquid-nitrogen-cooled, back-illuminated deep-depleted Symphony II CCD detector (1,024 × 256 pixels) and an Olympus BXFM microscope with a motorized XYZ sample stage. The spectral resolution was ~0.5 cm^−1^. A grating of 1,800 lines per mm and a confocal hole of 100 µm were used. A TopMode 405 laser source (Toptica Photonics) with a wavelength of 405 nm and a Torus 532 laser source (Laser Quantum) with a wavelength of 532 nm were used for excitation. Spectra were acquired in back-scattering geometry using an Olympus LMPlanFL N ×50 long-working-distance objective with a numerical aperture of 0.50 and a working distance of 10.6 mm. The spectrometer was calibrated daily using the 521 cm^−1^ line of silicon.

Three sets of heating experiments were run in which SFIs entrapped in a quartz chip were heated using a Linkam TS1500 heating–freezing stage operated by a TMS 94 Controller (Extended Data Fig. 1e). The stage was calibrated using the melting temperature of gold at 1,064 °C, and the accuracy of calibration was checked by melting KNO_3_ at 334 °C and NaCl at 801 °C.

In the first set of preliminary experiments, the aim was to establish the best strategy for the study of sulfur speciation (for example, to find the ideal inclusion size and depth, and heating rate) and find the approximate stability range of individual sulfur species, including sulfur radicals.

In the second set of experiments, we aimed to rapidly collect data on sulfur speciation in the SFIs at their entrapment temperature of 875 °C to prevent potential oxidation of the SFIs at high temperatures during analysis (Supplementary Figs. 6–8). Raman spectra of the liquid and vapour phases present in the SFIs were collected along with spectra of the host quartz at 25 °C, followed by heating to their entrapment temperature of 875 °C. A heating rate of 10 °C min^−^^1^ was applied, which was slow enough to prevent the sudden decrepitation of the fluid inclusion or the explosion of the entire quartz chip that we observed during preliminary heating experiments using higher heating rates. After keeping the SFIs at 875 °C for 10 min, spectra of the supercritical fluid phase and the host quartz were collected. Two sets of Raman spectra were collected for each phase (liquid, vapour, supercritical fluid and host quartz) for excitation at 405 nm and 532 nm, respectively. In each set, two accumulations of 30 s each were taken in the spectral window of 50–4,000 cm^−1^ first. In addition, five accumulations of 30 s each were taken in the spectral windows of 100–1,400 and 2,400–2,700 cm^−1^, where bands of the sulfur species occur.

In the third set of experiments, the aim was to monitor the changes in sulfur speciation during heating. Raman spectra of the host quartz and SFI phases except for that of solid sulfur (liquid and vapour below the homogenization temperature or supercritical fluid above the homogenization temperature) were collected at 25, 200, 300, 400, 500, 700 and 875 °C. A heating rate of 10 °C min^−1^ was applied. The homogenization temperature, which was marked by bubble disappearance, was always in the 450 ± 20 °C range in experiments with fluids containing 1 mol NaCl per kg H_2_O. In the case of the NaCl/KCl-free fluid the homogenization temperature was ~380 °C; for the fluid containing 1 mol KCl per kg H_2_O it was ~500 °C; and for fluids containing 4 mol NaCl per kg H_2_O or 4 mol KCl per kg H_2_O, homogenization took place just below the entrapment temperature of 875 °C. In some cases, spectra were also collected just below and above the homogenization temperature. The reactions between sulfur species in aqueous fluids have been found to be rapid in previous studies^18^. Nevertheless, the temperature was maintained for 10 min at each step to reach thermal and chemical equilibrium before to data collection. The attainment of equilibrium was confirmed at 400 °C (a moderate temperature with relatively slow reaction rates) via the collection of two spectra of the liquid phase in the same inclusion: one after 10 min and another after 70 min (Supplementary Fig. 13). Most spectra were collected with excitation at 405 nm. Under conditions where S_3_^−^ was observed during the first or second set of experiments, the spectra of all phases were also collected with excitation at 532 nm. In this set of experiments, two accumulations of 30 s each were taken in the spectral window of 50–4,200 cm^−1^ first. In addition, ten accumulations of 30 s each were taken in the spectral window of 350–700 cm^−1^, where sulfur radical species had previously shown strong signals.

To check whether the SFIs suffered any oxidation due to hydrogen loss and hence a change of sulfur speciation during heating before Raman analysis, first we collected room-temperature spectra of the SFI phases before and after heating. Whereas spectra collected before and after heating to 750 °C showed no change in speciation, in the case of heating to 875 °C we observed very minor changes. To measure the extent of oxidation of fluids entrapped at 875 °C, we collected spectra of the supercritical fluid phase at 750 °C, then continued the heating to 875 °C, left the SFIs there for twice the time of usual spectrum collection, then cooled it to 750 °C and collected another spectrum of the supercritical fluid phase at 750 °C (Supplementary Fig. 14). During our tests, 0.7% of the total sulfur content of the SFIs was oxidized from S^2−^ to S^4+^ in the SFIs entrapped at 875 °C. This means that during our experimental runs, where SFIs are kept above 750 °C for only half the time of our tests, 0.4% of the total sulfur content of the SFIs gets oxidized from S^2−^ to S^4+^ during the heating and analysis of SFIs entrapped at 875 °C. Accordingly, our speciation results were corrected for this oxidation effect.

### Quantification of sulfur speciation

To obtain quantitative data on sulfur speciation in aqueous fluids at their entrapment temperature of 875 °C, the integrated peak areas of different sulfur species normalized to the peak area of an internal reference—for example, the bending peak of H_2_O—may be used^68^. To be comparable to experimental run products, reference materials used for calibration should be (1) similar in nature to the run products, (2) analysed using the same instrument and (3) analysed under the same analytical conditions. For accurate quantification, the effect of density and salinity on the bands of the aqueous fluid must also be considered. For instance, with increasing NaCl concentration, both the SO_4_^2−^/H_2_O bending band ratios and the SO_4_^2−^/H_2_O stretching band ratios decrease, and the band shape in the H_2_O stretching mode region changes^69^.

To circumvent the potential inconsistencies outlined above, we decided to use the endmembers of our experimental run products as reference materials for calibration. In the experiment conducted at the second highest $f_{{\mathrm{O}}_2}$ (NNO + 2.6), SO_2_ is the only major sulfur species present in the supercritical fluid, and in the experiment conducted at the lowest $f_{{\mathrm{O}}_2}$ (NNO − 0.9), H_2_S and HS^−^ dominate with negligible amounts of SO_2_, close to the detection limit of Raman spectra. We used the stretching rather than bending peak of H_2_O as an internal reference, because the stretching peak has a much larger intensity in low-density supercritical fluids than the bending peak. Given that the reference materials are endmembers of our experimental run products, the shape of the stretching peak of H_2_O will be affected by temperature, pressure and salinity in the exact same way across our $f_{{\mathrm{O}}_2}$ experimental series, enabling the stretching peak to be used for normalization without further corrections of its intensity.

### Correction of Raman spectra and peak fitting for quantification of sulfur speciation

Using a liquid-nitrogen-cooled detector and a 405 nm laser enabled the collection of Raman spectra with a very high signal-to-noise ratio and a linear background, even at 875 °C (Extended Data Fig. 2). The *ν*_1_(HS^−^) and *ν*_1_(H_2_S) peaks were therefore fitted in the 405 nm spectra (Supplementary Fig. 15). However, owing to the overlap of the *ν*_1_(SO_2_), *ν*_1_′(SO_2_) and 2*ν*_1_(S_2_^−^) peaks in the 405 nm spectra (Supplementary Fig. 16), the 532 nm spectra that are void of the 2*ν*_1_(S_2_^−^) peak were used to fit the *ν*_1_(SO_2_) and *ν*_1_′(SO_2_) peaks. In each case, collecting the spectrum of the quartz host, conducted before the collection of each fluid spectrum at the same focal plane and ~10 µm away from the outer walls of the fluid inclusion, enabled subtraction of the quartz spectrum and isolation of the bands of water and aqueous species. Peak fitting was then performed on the residual fluid spectra using OriginPro software to obtain areas (integrated intensities) of the relevant Raman peaks. The area of the water stretching band was determined by integrating its intensity throughout the 3,200–3,800 cm^−1^ region.

The H_2_S/HS^−^ peak area ratios were similar across the investigated $f_{{\mathrm{O}}_2}$ range. To quantify the H_2_S/HS^−^ molar ratio, we used the relative molar scattering factors (*J*) of these sulfur species. The *J*(H_2_S)/*J*(HS^−^) ratio of ~1.5 shows little sensitivity to the applied laser wavelength^26^. Minor H_2_S_*n*_ species also occurred in the most reduced experiments and were quantified assuming *J*(H_2_S)/*J*(H_2_S_*n*_) = 1. These relative molar scattering factors were also considered for the quantification of individual sulfur species. Finally, our speciation results were corrected for the very minor effect of oxidation at magmatic temperatures (see ‘Raman spectroscopy’ in the Methods).

### Laser ablation inductively coupled plasma mass spectrometry

Analyses via LA-ICP-MS were carried out using an ESL 193 HE laser ablation system coupled with an Agilent 8900 triple quadrupole mass spectrometer at the Department of Earth Sciences, University of Geneva. Helium was used as the carrier gas at a flow rate of ~850 ml min^−1^. The instrument was tuned to ThO/Th < 0.3%, mass-21/mass-42 ≈ 0.2% and ^238^U^/232^Th ≈ 1.0 using the glass reference material NIST-610. The quartz-hosted fluid inclusions, previously not heated for Raman analysis, were ablated at a laser energy density of ~18 J cm^−2^ and a repetition rate of 10 Hz. A stepwise opening of the laser beam from 10 to 40 μm, depending on the size of the inclusion, was used^70^. On-peak dwell times were set to 10 ms for ^23^Na, ^27^Al and ^29^Si, and to 40 ms for ^197^Au. The glass reference material NIST-610 was used as an external standard, whereas the concentration of sodium in the experimental starting solution was used as an internal standard for data quantification. Each analysis block consisted of approximately 15–20 analyses of unknowns bracketed by two measurements at the beginning and end of each block of the NIST-610 standard. In each sample, 5–10 fluid inclusions were analysed. Data reduction was performed using the SILLS software^71^.

### Thermodynamic model calculations

Our experimental data on sulfur speciation were compared with data calculated using the HCh software package that enables free-energy minimization in complex aqueous fluid systems that rely the Helgeson–Kirkham–Flowers equation of state for aqueous species^72^. The list of chemical species that was considered for our system file along with the references for the thermodynamic data sources are summarized in Supplementary Table 5. Sulfur speciation was calculated in an aqueous fluid phase containing 1 mol NaCl per kg H_2_O and 5 mol% sulfur that corresponded to our experimental fluid, and under the pressure and temperature conditions that covered our experimental set-up, that is, at 2,000 bar, 875 °C and in $0.5 \log f_{{\mathrm{O}}_2}$ steps ranging from NNO − 1.5 to NNO + 3.5. The $f_{{\mathrm{O}}_2}$ of the system was defined in the presence of water by considering H_2_ as a perfectly mobile component^73^.

In addition, we ran calculations to address gold solubility under the same conditions. For these, the input file contained 1 mol Au per kg H_2_O. The list of gold species considered for the system file can be found in Supplementary Table 5.

## Online content

Any methods, additional references, Nature Portfolio reporting summaries, source data, extended data, supplementary information, acknowledgements, peer review information; details of author contributions and competing interests; and statements of data and code availability are available at 10.1038/s41561-024-01601-3.

## Supplementary information

Supplementary Information Supplementary Tables 1–8, Figs. 1–17 and text.

Supplementary Data 1 Source data for Supplementary Figs. 1–5, 7, 8 and 10–17.

## Source data

Source Data Fig. 1 Raw Raman spectra.

Source Data Fig. 2 Experimental data points and simulation results.

Source Data Fig. 3 Raw Raman spectra.

Source Data Fig. 5 Experimental data points and simulation results.

Source Data Extended Data Fig. 2 Calculations and raw Raman spectra.

Source Data Extended Data Fig. 3 Diffuse reflectance spectrum.

## Data Availability

The data that support the findings of this study, including raw Raman spectra and LA-ICP-MS data, are available via figshare at 10.6084/m9.figshare.27215496 (ref. ^74^). Source data are provided with this paper.
