# Supplementary material for: Sulfur species and gold transport in arc magmatic fluids
Source: Nat Geosci. 2024 Dec 16;18(1):98–104. doi: 10.1038/s41561-024-01601-3 (PMC11732748; doi:10.1038/s41561-024-01601-3)
Supplement: Supplementary file 1 — Supplementary Tables 1–8, Figs. 1–17 and text. [file 41561_2024_1601_MOESM1_ESM.pdf]

nature geoscience

Supplementary information https://doi.org/10.1038/s41561-024-01601-3

Sulfur species and gold transport in arc magmatic fluids

In the format provided by the authors and unedited

Supplementary tables

Study	Laser wavelength (nm)	Reported sulphur radical ions
Pokrovski and Dubrovinsky (2011)	632	S_3^-
Jacquemet et al. (2014)	532.1<br>637.7	S_3^-
Pokrovski and Dubessy (2015)	457.9<br>514.5	S_2^-, S_3^-
Colin et al. (2020)	473<br>532	S_2^-, S_3^-

Table S1. Laser wavelengths employed in studies attempting the quantification of the S_3^- radical ion. Note that in all studies at least one laser wavelength used lies inside the absorbance band of the S_3^- ion (Extended Data Figure 1) and hence S_3^- was reported in all of them. However, in studies where excitations lying outside the absorbance band of the S_2^- ion were used only, this radical species remained unnoticed and was only reported in studies employing at least one excitation that lies inside its absorbance band. These observations suggest that in the absence of Raman resonance effect, the concentration of sulphur radicals may be below the detection limit of non-resonant Raman spectra of fluids at the physiochemical conditions of respective studies. Moreover, given that the absorption band of S_2^- is much narrower than that of S_3^- (Extended Data Figure 3), more, commonly used excitations can detect S_3^-, potentially leading to the biased detection of sulphur radicals in favour of S_3^-.

fO_2 (ΔNNO)	H_2S	HS^-	H_2S_n	S_3^-	SO_2	SO_4^{2-}	HSO_4^-
Experimental data, starting fluid composition: H_2O + 1 mol NaCl/kg H_2O + 5 mol% H_2SO_4							
2.6	B. d. l.	B. d. l.	B. d. l.	B. d. l.	100.0	B. d. l.	B. d. l.
2.1	2.5	3.7	B. d. l.	B. d. l.	93.8	B. d. l.	B. d. l.
1.6	2.5	4.4	B. d. l.	B. d. l.	93.1	B. d. l.	B. d. l.
1.1	6.0	14.4	B. d. l.	B. d. l.	79.5	B. d. l.	B. d. l.
0.6	11.3	21.6	B. d. l.	B. d. l.	67.2	B. d. l.	B. d. l.
0.1	19.3	33.9	B. d. l.	B. d. l., R. R.	46.8	B. d. l.	B. d. l.
-0.4	31.0	47.2	9.8	B. d. l., R. R.	12.1	B. d. l.	B. d. l.
-0.9	29.0	61.0	7.5	B. d. l., R. R.	2.6	B. d. l.	B. d. l.
Thermodynamic model calculations, starting fluid composition: H_2O + 1 mol NaCl/kg H_2O + 5 mol% H_2SO_4							
3.5	<0.1	<0.1	N. c.	<0.1	98.8	<0.1	1.1
3	0.1	<0.1	N. c.	<0.1	99.0	<0.1	0.8
2.5	0.6	<0.1	N. c.	0.5	98.3	<0.1	0.5
2	3.3	<0.1	N. c.	5.5	91.1	<0.1	0.1
1.5	13.7	<0.1	N. c.	19.3	67.0	<0.1	<0.1
1	35.4	<0.1	N. c.	33.5	31.1	<0.1	<0.1
0.5	56.1	<0.1	N. c.	33.8	10.1	<0.1	<0.1
0	73.2	<0.1	N. c.	24.8	2.0	<0.1	<0.1
-0.5	83.6	<0.1	N. c.	16.0	0.4	<0.1	<0.1
-1	90.5	<0.1	N. c.	9.4	0.1	<0.1	<0.1
-1.5	94.8	<0.1	N. c.	5.1	<0.1	<0.1	<0.1

Table S2. Experimental data on sulphur speciation in the supercritical fluid phase at 2 kbar and 875°C compared with thermodynamic model calculations. The calculations include thermochemical data for the S_3^- radical species of Pokrovski and Dubessy (2015). Concentration values refer to % of total sulphur. Note that calculations severely underestimate the concentration of HS^- and severely overestimate the concentration of S_3^- in the fluid. In our experiments, the concentration of sulphur radical species S_2^- and S_3^- remained below detection limit in their respective non-resonant Raman spectra at all investigated conditions. Abbreviations: B. d. l. = below detection limit, R. R. = observed in resonant Raman spectra, N. c. = species not considered due to missing thermochemical data.

fO_2 (ΔNNO)	Liquid phase, 25°C	Vapour phase, 25°C	Supercritical fluid phase, 875°C
Starting fluid composition: H_2O + 5 mol% H_2SO_4			
-0.3	H_2S, HS^-, SO_4^{2-}	H_2S, H_2	H_2S, HS^-, H_2S_n, SO_2, S_2^-
Starting fluid composition: H_2O + 1 mol NaCl/kg H_2O + 5 mol% H_2SO_4			
-0.4	H_2S, HS^-, SO_4^{2-}	H_2S, H_2	H_2S, HS^-, H_2S_n, SO_2, S_2^-, S_3^-
Starting fluid composition: H_2O + 4 mol NaCl/kg H_2O + 5 mol% H_2SO_4			
-0.4	H_2S, HS^-, SO_4^{2-}	H_2S, H_2	H_2S, HS^-, H_2S_n, SO_2, S_2^-, S_3^-
Starting fluid composition: H_2O + 1 mol KCl/kg H_2O + 5 mol% H_2SO_4			
-0.4	H_2S, HS^-, SO_4^{2-}	H_2S	H_2S, HS^-, H_2S_n, SO_2, S_2^-, S_3^-
Starting fluid composition: H_2O + 4 mol KCl/kg H_2O + 5 mol% H_2SO_4			
-0.4	H_2S, HS^-, SO_4^{2-}	H_2S	H_2S, HS^-, H_2S_n, SO_2, S_2^-, S_3^-

Table S3. Sulphur species and H_2 gas detected in SFI. Note that all SFI contained a solid sulphur crystal at 25°C. The indicated fO_2 values refer to the entrapment conditions of SFI at 2 kbar and 875°C.

fO_2 (ΔNNO)	ppm	n
2.6	129(±52)	5
2.1	91(±21)	6
1.6	147(±31)	6
1.1	388(±80)	5
0.6	500(±34)	4
0.1	912(±316)	8
-0.4	1390(±100)	6
-0.9	1420(±360)	7

Table S4. Gold solubility in our experimental fluids determined by LA-ICP-MS. The indicated *f*O_2 values refer to the entrapment conditions of SFI at 2 kbar and 875°C.

Mineral name or chemical formula	Reference
Solid species	
Quartz	Helgeson et al. (1978)
Sulphur	Chase (1998)
Aqueous species	
H_2O, H^+, OH^-, Na^+, K^+, Cl^-, KCl, SiO_2	Johnson et al. (1992)
NaOH, KOH, SO_3^{2-}	Shock et al. (1997)
HCl	Tagirov et al. (1997)
NaCl, $HSiO_3^-$	Sverjensky et al. (1997)
Na_2SiO_3	Sullivan et al. (2022)
H_2, O_2, H_2S, SO_2	Akinfiev and Diamond (2003)
HS^-, $S_2O_3^{2-}$, SO_4^{2-}, HSO_3^-, HSO_4^-	Shock and Helgeson (1988)
S_3^-	Pokrovski and Dubessy (2015)
Gaseous species	
H_2	Robie et al. (1995)
Gold species for gold solubility calculations	
Gold	Robie et al. (1995)
Au^+, AuOH, $Au(OH)_2^-$, AuCl, $AuCl_2^-$, AuHS, $Au(HS)_2^-$	Akinfiev and Zotov (2001)

Table S5. Species included in the HCh model calculations and references for the thermodynamic data sources.

Supplementary texts and figures

Rationale behind experimental fluid composition

For the *f*O_2 series experiments, a fluid composition closely analogous to those common in natural porphyry ore-forming systems was chosen with NaCl concentration of 1 mol/kg H_2O and 5 mol % (~7 wt%) sulphur, and with no HCl added. In terms of pH, or better say HCl/total metal chloride ratio, considering the high temperature of the experiments, this fluid would maintain equilibrium with metaluminous silicate melts, common in porphyry ore-associated magmatic systems, based on numerous published experimental studies addressing fluid – melt equilibrium (e.g., Williams et al. 1997, Hsu et al. 2019). The salinity of the fluid falls in the middle of the characteristic range of magmatic input fluids identified in high-temperature fluid inclusions in the roots of porphyry ore deposits (e.g., Heinrich 2005, Heinrich 2024). The employed sulphur concentration is characteristic of fluids that are in equilibrium with either sulphide or anhydrite saturated silicate melts at upper crustal pressures[20–22]. Magmatic anhydrite and sulphide are common in magmas associated with porphyry ore systems[19].

Reactions of sulphur species upon heating

Upon heating, the concentration of SO_4^{2-} in the liquid drops rapidly up to ~100°C and stays below the detection limit of spectra above 100°C (Figure S1) as observed previously[23]. Simultaneously, the concentration of HSO_4^- increases rapidly up to 100°C in accordance with the protonation reaction:

$SO_4^{2-}(aq) + H^+(aq) = HSO_4^-(aq)$ Equation S1

Above 100°C, the concentration of HSO_4^- starts to drop (Figure S1). The solid sulphur crystal melts and dissolves into the liquid and the homogenization of liquid and vapour phases takes place at 450±20°C. SO_2 in the liquid appears around 300°C and its concentration increases with further heating as noted previously[23], even after the dissolution of sulphur crystal. These observations are in perfect agreement with sulphur comproportionation reactions proposed by Drummond (1981). In the first, prevailing at lower temperatures, solid sulphur participates in the reaction:

$2HSO_4^-(aq) + S(s) + 2H^+(aq) = 3SO_2(aq) + 2H_2O(l)$ Equation S2

Once the sulphur crystal dissolves, the second sulphur comproportionation reaction becomes dominant:

$3HSO_4^-(aq) + H_2S(aq) + 3H^+(aq) = 4SO_2(aq) + 4H_2O(l)$ Equation S3

All reactions observed upon heating are reversible.

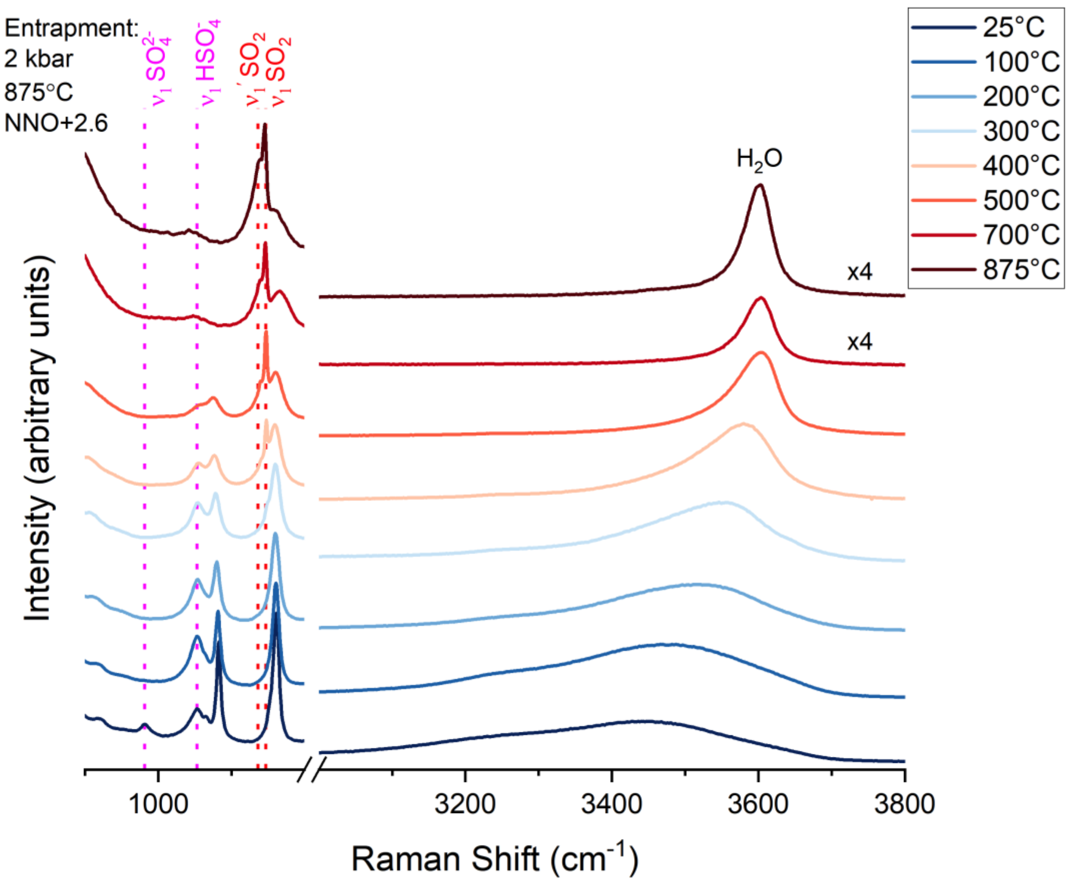

Figure S1. Changes in sulphur speciation in the liquid (25-400°C) and supercritical fluid (500-875°C) during heating as shown by raw Raman spectra. Note that the large SFI monitored in the heating experiment decrepitated above 500°C and spectra at 700 and 875°C were collected from two different, much smaller SFI.

Sulphur radical ions

The concentration of S_2^- and S_3^- radical ions remained below the detection limit of their respective non-resonant Raman spectra at all investigated conditions. Despite their miniscule concentrations, resonance Raman spectroscopy allows for detailed study of these enigmatic sulphur species and the differences between them. Whereas S_3^- is only present at reducing conditions, the stability field of S_2^- extends to much higher fO_2 conditions (Figure S2). Furthermore, below the homogenisation temperature, S_3^- was observed exclusively in the denser liquid phase with the strongest resonance Raman signal at 300°C. In contrast, S_2^- forms in the vapour phase too and shows the strongest resonance Raman signal in the supercritical fluid at 500°C.

Our observations suggest that the density and ionic strength of the fluid phase and concentration of sulphur control the stability of sulphur radicals. In a liquid, sulphur radicals can form directly, through the temperature-induced gradual dissociation of S_8 sulphur rings. First, S_n^{2-} polysulphide dianions (e.g., S_6^{2-}, S_4^{2-}) form, followed by their dissociation to form sulphur radicals (e.g., S_3^-, S_2^-)[25]. The gradual dissociation of S_8 sulphur rings to polysulphides and then to sulphur radicals is consistent with the observation of polysulphides up to 300°C and sulphur radicals up to their maximum experimental temperature of 450°C by Pokrovski and Dubrovinsky (2011). Note that their experimental fluids were denser and contained more sulphur. Consequently, a lot of the sulphur was still present in the form of longer sulphur chains.

Sulphur radicals can also form indirectly, through the ionization of short sulphur chains in the less dense vapour and supercritical fluid. Just below the homogenisation temperature, we observe a much stronger S_2^- signal (but not S_3^-) in the vapour phase than in the liquid phase

(Figure S3). This is because the ionization of short sulphur chains is more effective in the vapour phase, in which an electron can be readily transferred to a small sulphur molecule[25].

The only cations capable to charge balance and stabilise sulphur radicals in our experiments are H^+ and Na^+. Pokrovski and Dubessy (2015) argue that the formation of S_3^- is controlled by the concentration of H^+, i.e. pH:

$2H_2S(aq) + SO_4^{2-}(aq) + H^+(aq) = S_3^-(aq) + 0.75O_2(g) + 2.5H_2O(l)$ Equation S4

However, thermodynamic model calculations using thermochemical data of Pokrovski and Dubessy (2015) not only severely overestimate the concentration of radical species but are also inconsistent with their proposed equilibrium. The calculations predict very low amounts of S_3^- in a NaCl-free fluid, in which the pH is predicted to be lower than in NaCl-rich fluids (Figure S4). Moreover, at a given salinity, the concentration of S_3^- seems to correlate very well with the concentration of Na^+ ions in the fluid and suggests that Na^+ ions play a much more important role in stabilising sulphur radicals than H^+ ions.

In ultramarines, S_3^- is strongly stabilized by Na^+ ions[26]. To study the effect of Na^+ concentration on the formation and stabilization of S_3^- in magmatic fluids, we conducted additional experiments using a NaCl-free and a 4 mol NaCl/kg H_2O fluid, both having the same (5 mol%) sulphur concentration and run these at ~NNO-0.4, where the maximum S_3^- concentration was observed in the 1 mol NaCl/kg H_2O fluids (Table S3)(Figure S5). The signal of S_3^- in the resonant Raman spectra was gradually increasing from almost invisible in the NaCl-free fluid to very prominent in the 4 mol NaCl/kg H_2O fluid, suggesting that in a system open for redox exchange (e.g., H_2 loss), Na^+ ions play an essential role in charge balancing S_3^- according to:

$6H_2S + 2Na^+(aq) + 2Cl^-(aq) = 2S_3^-(aq) + 2Na^+(aq) + 2HCl + 5H_2$ Equation S5

In our fluids, S_3^- is most likely stabilised as Na^+–S_3^- ion pairs.

To see whether a different alkali ion, K^+ can stabilise S_3^-, we also run experiments with fluids containing 1 and 4 mol KCl/kg H_2O instead of NaCl (Table S3)(Figure S5). Increasing KCl concentration too led to the enhancement of S_3^- signal, most likely via the formation of K^+–S_3^- ion pairs. In conclusion, both Na^+ and K^+ alkali ions successfully stabilise S_3^- in high-pressure-temperature fluids. However, no obvious trend between salinity and the formation of S_2^- and was observed.

a

b

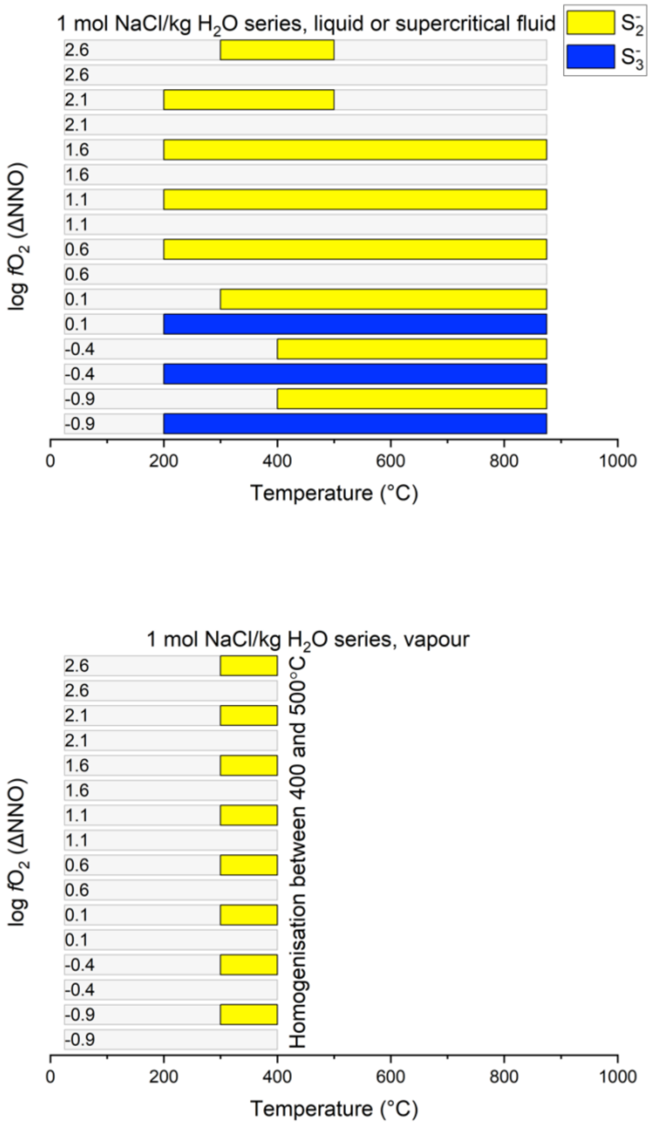

Figure S2. Stability fields of sulphur radical species based on the detection of S_2^- and S_3^- in their resonant Raman spectra in the 1 mol NaCl/kg H_2O series: in liquid or supercritical fluid (a) and in vapour (b). Note that both S_3^- and S_2^- remained below detection limit in their respective non-resonant Raman spectra at all investigated conditions. The indicated fO_2 values refer to the entrapment conditions of SFI at 2 kbar and 875°C.

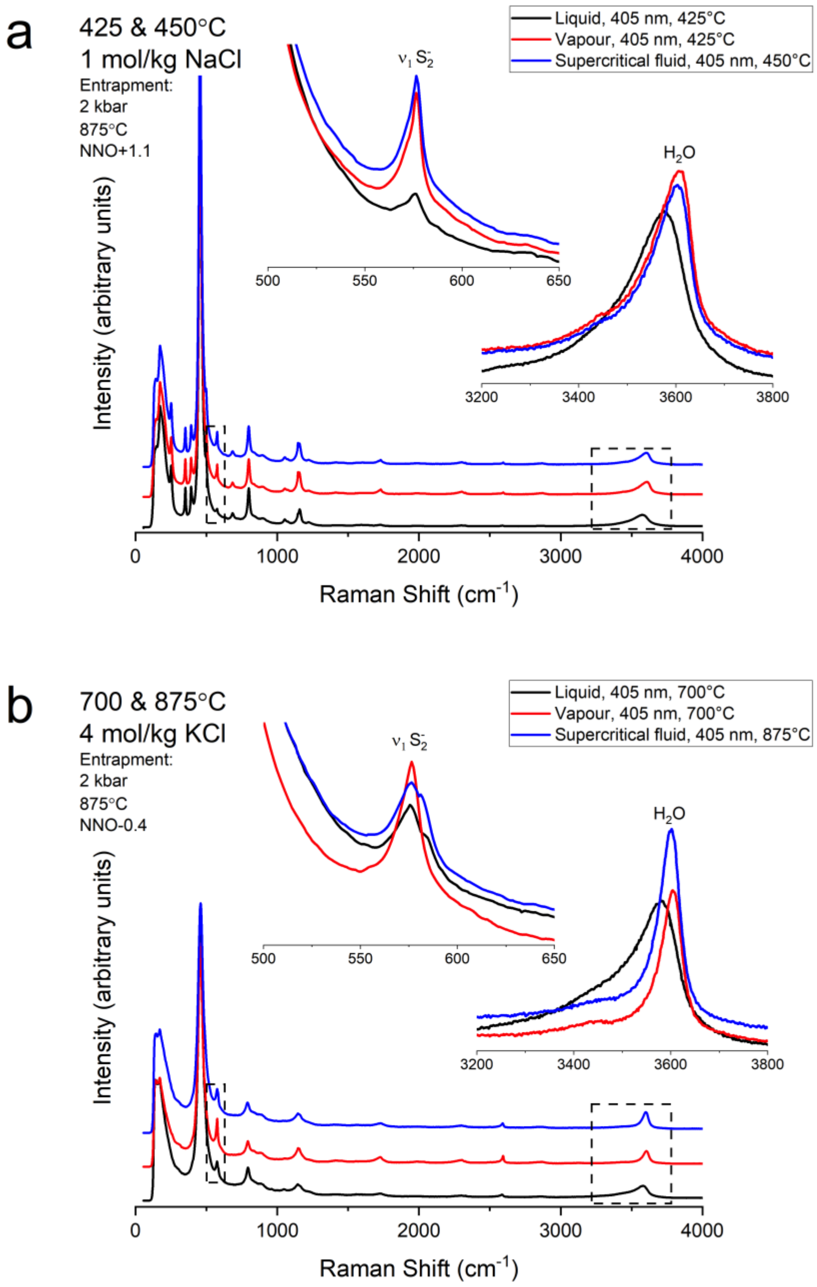

Figure S3. Raw Raman spectra of the liquid and vapour phases collected at a temperature just below homogenization and the supercritical fluid phase collected just above homogenization in a SFI. Note that in both fluids the S_2^- signal is higher in the vapour than liquid phase. Spectra were vertically offset for better readability (except those in the insets).

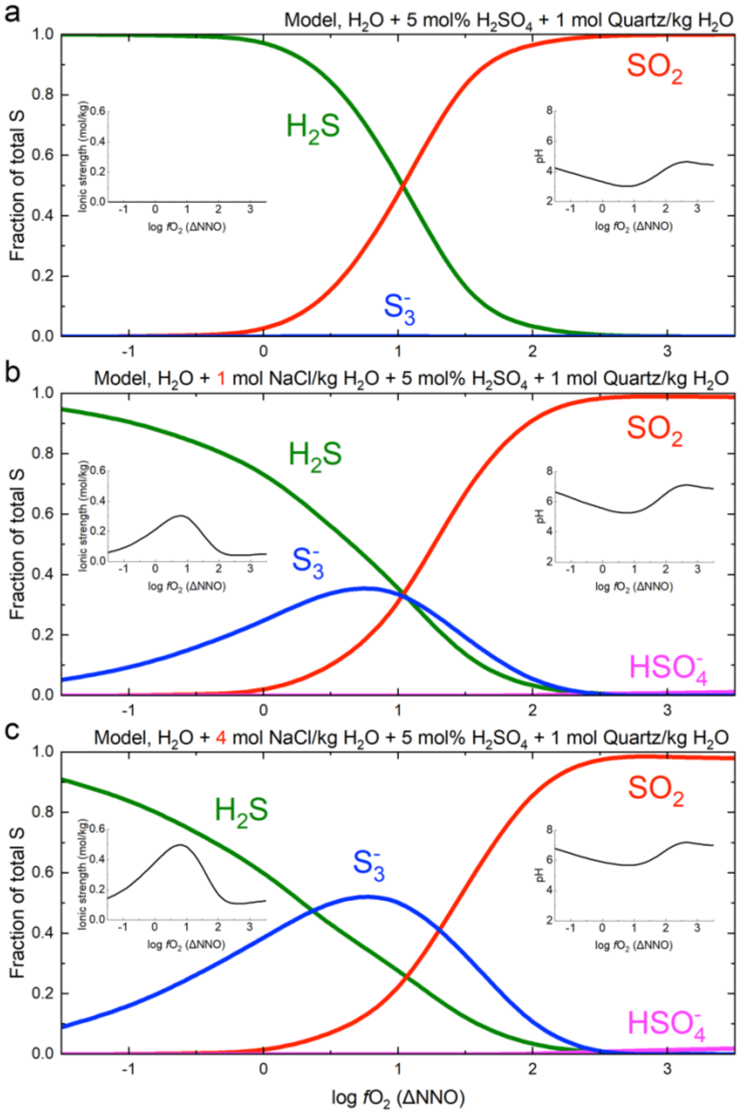

Figure S4. Sulphur speciation in aqueous supercritical fluid phases of different NaCl concentrations at 2 kbar and 875°C, calculated using thermochemical data reported in Table S5 and data for the S_3^- radical[3] included in the calculation. Only those sulphur species are visualised that reached a concentration of at least 1% of total sulphur at any of the considered fO_2 values. Note the relationship between ionic strength and the concentration of S_3^- radicals. Also note that these calculations predict over 30% total sulphur present as S_3^- at some of our experimental conditions. In contrast, S_3^- stayed below detection limit of its non-resonant spectra at all investigated conditions.

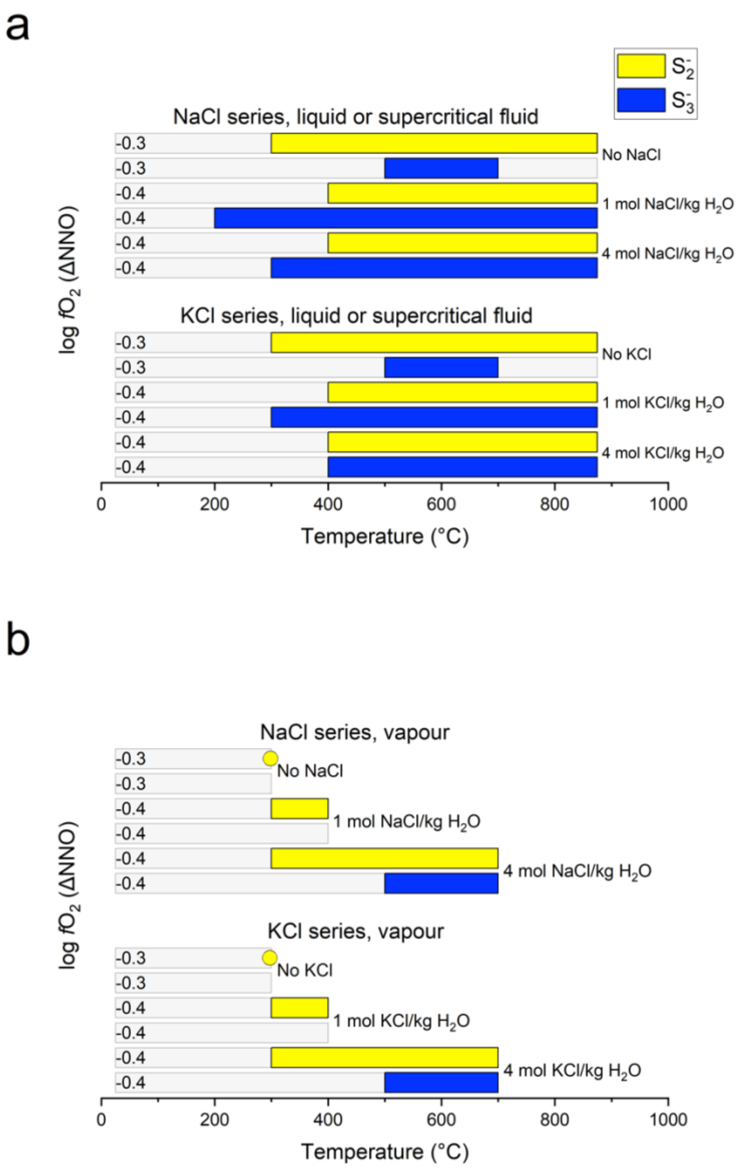

Figure S5. Stability fields of sulphur radical species based on the detection of S_2^- and S_3^- in their resonant Raman spectra in the salinity series: in liquid or supercritical fluid (a) and in vapour (b). The indicated fO_2 values refer to the entrapment conditions of SFI at 2 kbar and 875°C. Homogenization occurs between 300 and 400°C in the NaCl and KCl-free experiments, between 400 and 500°C in the experiments containing 1 mol NaCl/kg H_2O or 1 mol KCl/kg H_2O, and between 700 and 800°C in the experiments containing 4 mol NaCl/kg H_2O or 4 mol KCl/kg H_2O. Note that the addition of NaCl or KCl extends the stability field of S_3^-.

Brine condensation

In contrast to the room temperature sulphur speciation of SFI, where sulphate species HSO_4^- and SO_4^{2-} dominate the oxidising end of investigated fO_2 conditions (Figure 1c)[27], only SO_2 was detected in the most oxidised supercritical fluid at magmatic temperatures (Figure 1d). However, in fluids entrapped at NNO+2.6, +2.1, and +1.6, shortly after reaching their entrapment temperature of 875°C, HSO_4^- with concentrations just above the detection limit of Raman spectra appeared. Approximately 1 h after reaching 875°C, a SO_4-rich, high-density brine started to condense from the supercritical fluid due to the density drop associated with water diffusion and due to oxidation associated with hydrogen diffusion from the SFI at magmatic temperatures (Figures S6 and S7). Sulphur transfer to the brine can be described by:

$SO_2(aq) + 2H_2O + 2Na^+(aq) + 2Cl^-(aq) = SO_4^{2-}(aq, brine) + 2Na^+(aq, brine) + 2HCl(aq) + H_2(aq)$ Equation S6

Given the lack of H_2SO_4, SO_4^{2-} in the brine is likely balanced by Na^+ or other alkalis. In magmatic systems, these can originate from the melt or minerals of the wall rock:

$SO_2(aq) + Na_2O(m) + H_2O = SO_4^{2-}(aq) + 2Na^+(aq) + H_2(aq)$ Equation S7

Equivalent equations can be written with K^+ and other alkalis, e.g.:

$SO_2(aq) + 2H_2O + 2K^+(aq) + 2Cl^-(aq) = SO_4^{2-}(aq, brine) + 2K^+(aq, brine) + 2HCl(aq) + H_2(aq)$ Equation S8

$SO_2(aq) + K_2O(m) + H_2O = SO_4^{2-}(aq) + 2K^+(aq) + H_2(aq)$ Equation S9

To check the timescale and extent of SFI oxidation, we collected room temperature spectra of SFI containing both reduced and oxidised sulphur species before heating the inclusion, after keeping it at 875°C for ~0.5 h, and after reheating and keeping it at 875°C for ~8 h. In addition, we also collected high temperature spectra of the supercritical fluid over a period of ~8 h. While keeping the SFI at 875°C for ~0.5 h, a time required for heating, thermal

and chemical equilibration, and spectrum collection at magmatic temperatures, had negligible effect on sulphur speciation, ~8 h was a period long enough to oxidise a significant part of the sulphur content (Figure S8). To investigate sulphur speciation at extremely oxidising conditions, we entrapped both NaCl-bearing and NaCl-free fluids at NNO+7.4 (see next section).

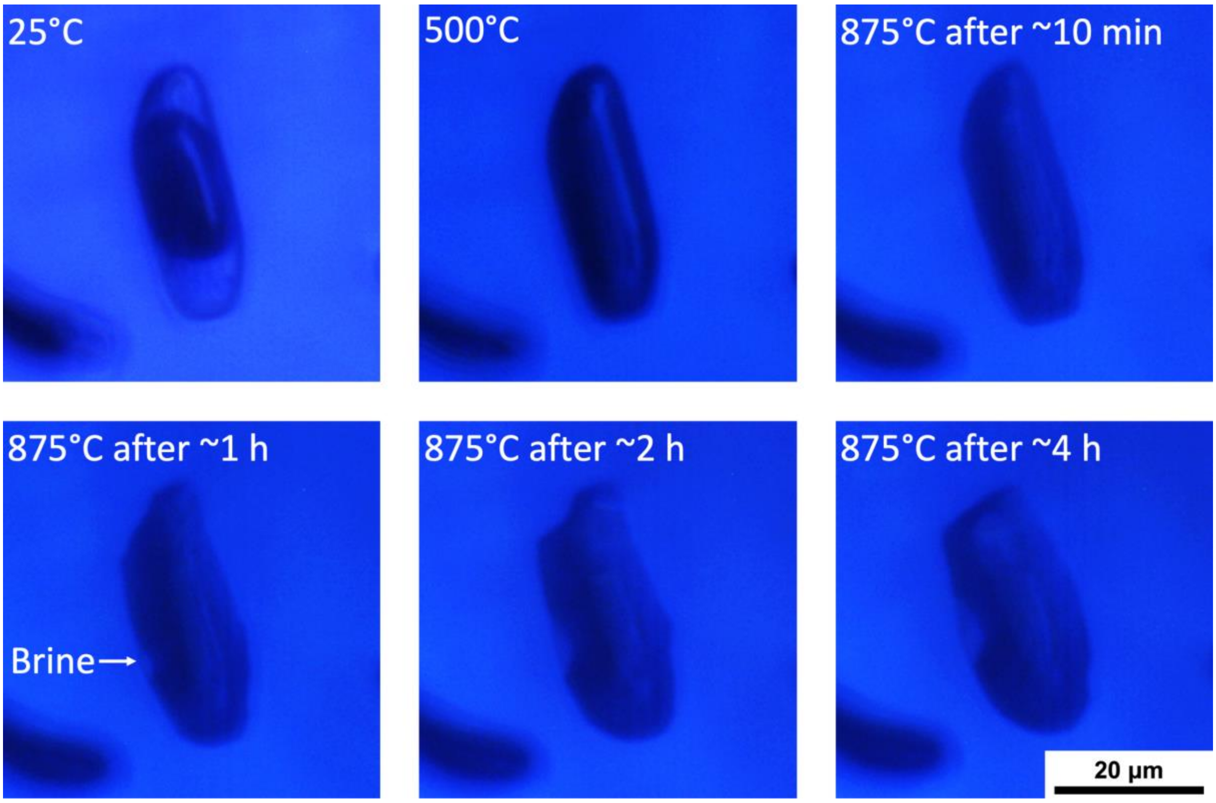

Figure S6. Evolution of phases with temperature in a SFI entrapped at 2 kbar, 875°C, and NNO+1.60. At 500°C, ~50°C above the homogenization temperature, only a supercritical fluid phase is present. Approximately 1 h after reaching 875°C, a SO_4-rich high-density brine starts to separate and gradually increases in volume with time.

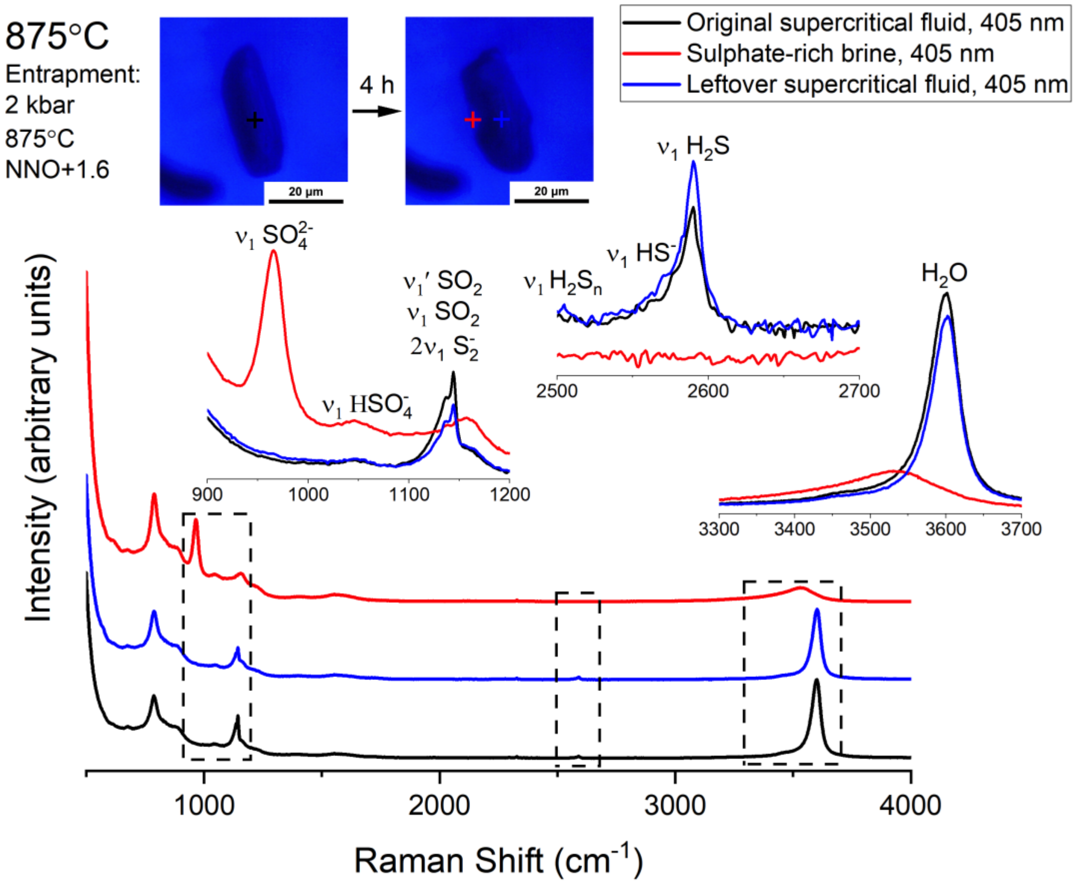

Figure S7. Raw Raman spectra of the supercritical fluid phase collected 10 minutes after reaching 875°C, the SO_4-rich high-density brine that separated from it and the leftover supercritical fluid, both collected 4 h after reaching 875°C. Note that the concentration of sulphide species in the supercritical fluid slightly increased upon brine condensation suggesting that besides sulphur oxidation, some SO_2 disproportionation also takes place. Spectra were vertically offset for better readability (except those in the insets).

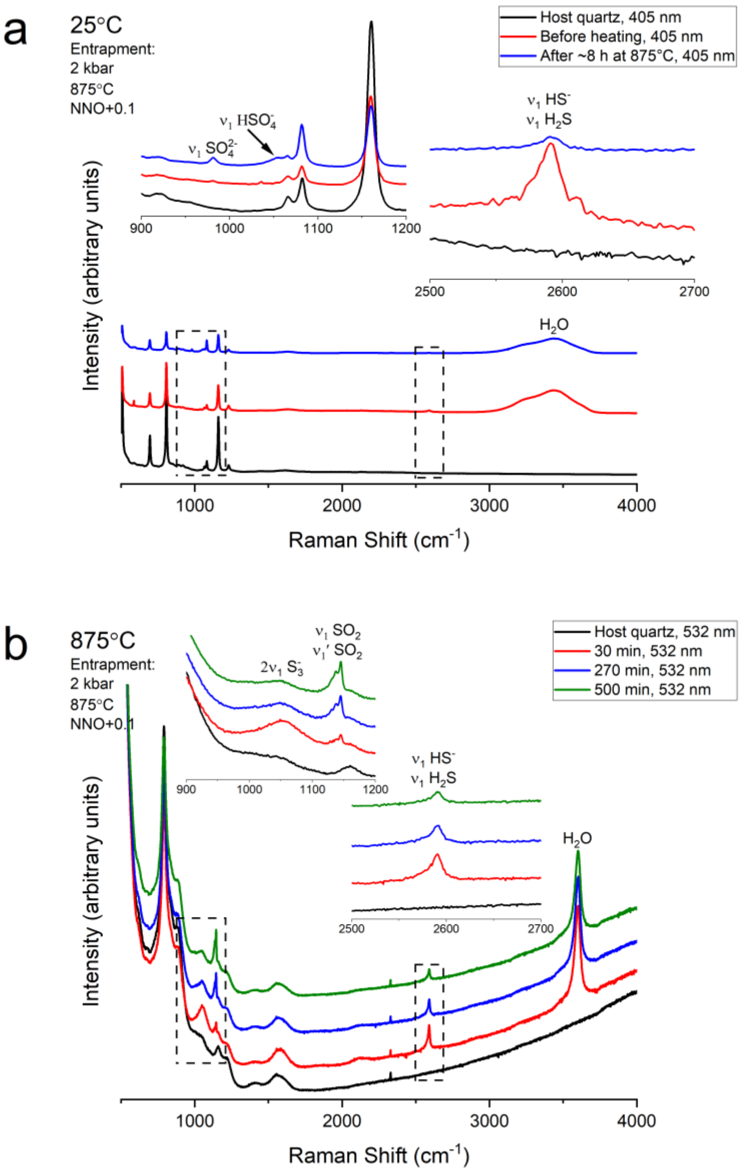

Figure S8. Oxidation of SFI at high temperature: a) raw 405 nm Raman spectra of the fluid phase collected at 25°C before and after keeping the SFI at 875°C for 8 h. Note that the $\nu_1(SO_4)/\nu_1(H_2S)$ and $\nu_1(HSO_4^-)/\nu_1(H_2S)$ band ratios increase substantially after keeping the inclusion at 875°C for ~8 h. b) raw 532 nm Raman spectra of the supercritical fluid phase collected at 875°C. Note that during oxidation of SFI, the area of peaks of sulphide species and S_3^- decrease while those of SO_2 increase. Spectra were vertically offset for better readability.

Sulphur speciation at extremely oxidising conditions

To investigate sulphur speciation at extremely oxidising conditions, we entrapped a supercritical fluid at NNO+7.4. We observed that the relative size of sulphur crystal in the quenched SFI of supercritical fluid entrapped at NNO+7.4 is smaller than in case of a supercritical fluid entrapped at NNO+2.6 (Figure S9). The heating of these SFI resulted in the rapid condensation of a brine above 600°C, well below the target temperature of 875°C. In contrast, the condensation of brine from the fluid entrapped at NNO+2.6 started only ~1 h after reaching the target temperature of 875°C. This behaviour can be explained by the presence of S^{6+} species in the supercritical fluid entrapped at NNO+7.4. Indeed, HCh calculations suggest that approximately 10% of total sulphur should be present as HSO_4^- at NNO+7.4. Due to the presence of S^{6+} species, the concentration of SO_2 is lowered, which leads to the precipitation of a smaller sulphur crystal due to sulphur disproportionation upon quenching. The presence of S^{6+} species also explains the rapid condensation of a Na- and sulphate-rich brine, which does not require the oxidation of already oxidised S^{6+} sulphur. To find whether S^{6+} species form in the absence of Na, we entrapped a fluid void of NaCl at the same experimental conditions. Besides SO_2, HSO_4^- is clearly present at NNO+7.4 in the NaCl-free supercritical fluid (Figure S10). However, it is very unlikely that such extremely oxidising conditions can exist in nature. In magmatic fluids entrapped at 875°C and geologically relevant fO_2 conditions, HSO_4^- remains below the detection limit of Raman spectra.

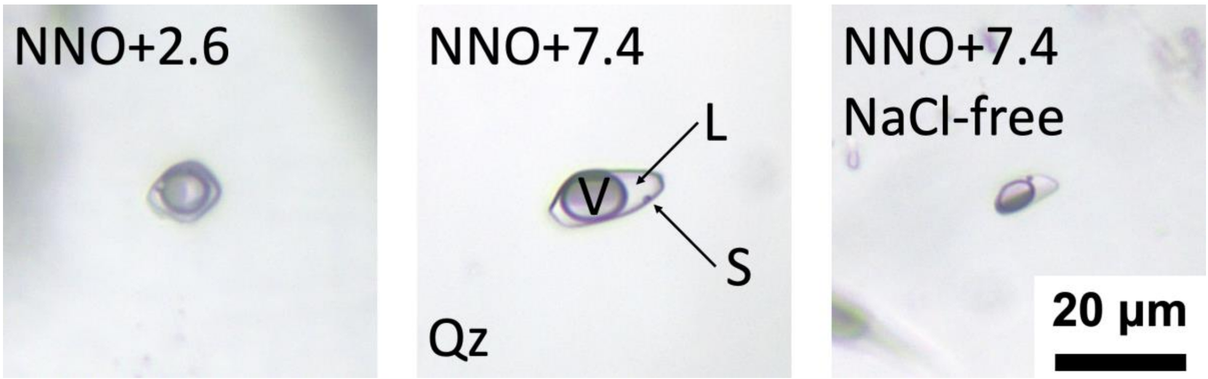

Figure S9. Plane polarized transmitted light microscope images (photomicrographs) of SFI trapped in quartz (Qz) host with a liquid phase (L), a vapour bubble (V), and a sulphur crystal (S). Both NaCl-bearing and NaCl-free fluids were entrapped at 2 kbar, 875°C and fO_2 of NNO+7.4. Note that the relative size of sulphur crystal in SFI entrapped at NNO+7.4 is smaller than in SFI entrapped at NNO+2.6.

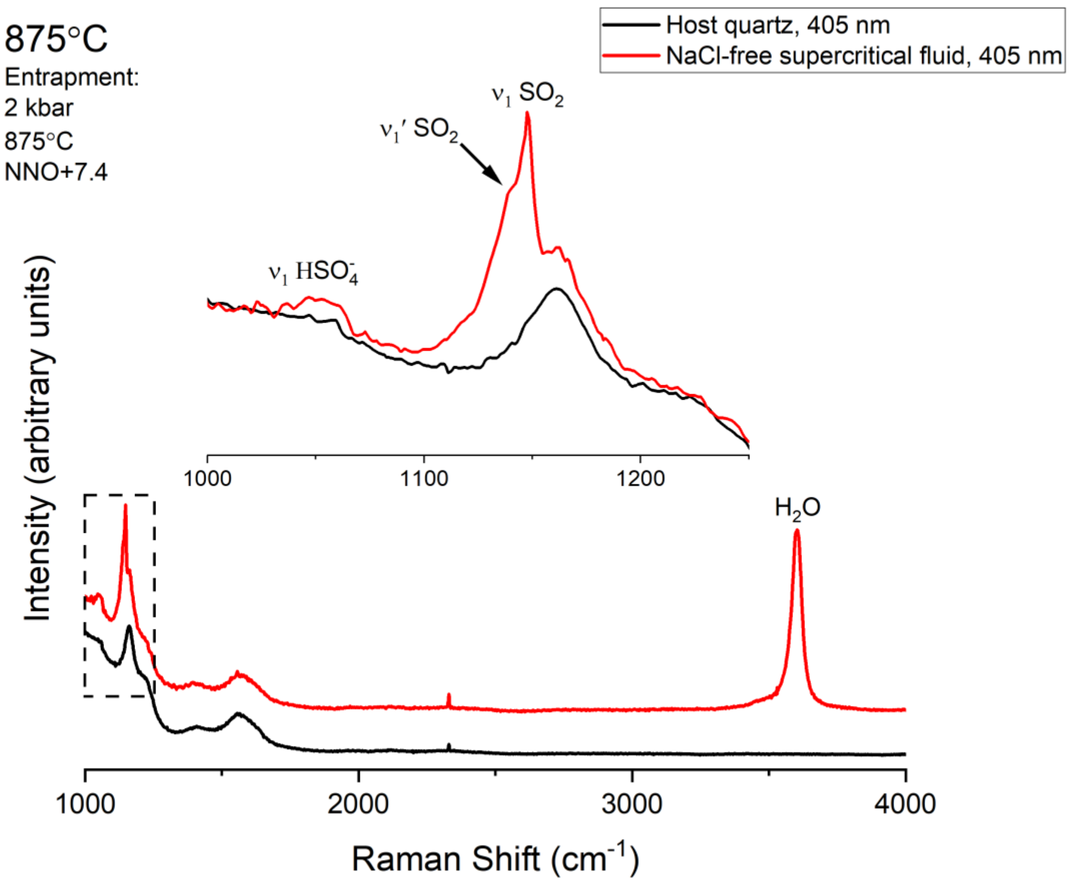

Figure S10. Raw 405 nm Raman spectrum of the NaCl-free supercritical fluid phase and an intensity normalized 405 nm Raman spectrum of the host quartz. Spectra were vertically offset for better readability (except those in the inset).

Assignment of the ~1137 cm^{-1} peak

The ~1137 cm^{-1} peak appears around 400°C as a shoulder to the ~1144 cm^{-1} peak corresponding to the S=O stretching band of SO_2 (Figure S1)[23,28]. Upon further heating, its area increases while that of the ~1144 cm^{-1} peak simultaneously decreases (Figure S1)[23,28]. The ~1137 cm^{-1} peak was interpreted to be the S=O stretching band of H_2SO_4 [23,28].

We present three lines of evidence that the ~1137 cm^{-1} peak is instead arising from the splitting of S=O stretching band of SO_2. First, we observed that at constant temperature of 875°C, the ratio of the areas of the ~1137 cm^{-1} peak and the ~1144 cm^{-1} peak corresponding to the S=O stretching band of SO_2 (A_{1137}/A_{1144}) remains constant regardless of fO_2. Indeed, in supercritical fluids dominated by sulphide species entrapped at NNO+0.1 the A_{1137}/A_{1144} ratio is the same as in supercritical fluid free of sulphide species entrapped at NNO+2.6 (Figure S11). If the ~1137 cm^{-1} peak belonged to H_2SO_4, the A_{1137}/A_{1144} ratio would have increased with increasing fO_2. However, the A_{1137}/A_{1144} remained the same even in the supercritical fluid entrapped at NNO+7.4 (Figure S11). Second, in a Raman heating experiment, where a relatively reduced fluid was kept at 875°C for ~8 h to investigate the effect of oxidation, the area of sulphide (H_2S and HS^-) peaks was gradually decreasing, while the areas of ~1137 cm^{-1} and ~1144 cm^{-1} peaks increased simultaneously, while the I_{1137}/I_{1144} ratio remained constant (Figure S8b). In case of a relatively oxidised fluid, upon formation of a sulphate-rich brine, the areas of ~1137 cm^{-1} and ~1144 cm^{-1} peaks decreased simultaneously, while the A_{1137}/A_{1144} ratio remained constant and the same as in the previous experiment (Figure S7). Once again, if the ~1137 cm^{-1} peak belonged to H_2SO_4, the A_{1137}/A_{1144} ratio would have been different at these contrasting fO_2 conditions and would have changed during oxidation at 875°C. Finally, the highest temperature spectrum of H_2SO_4 available in literature and collected at 513°C shows three peaks centred at ~858, ~1054, and ~1186 cm^{-1} [29]. Of these three, the weakest peak at

~1054 cm^{-1} is produced by HSO_4^- diminishing with increasing temperature and only the highest intensity peak at ~858 cm^{-1} and the second highest intensity ~1186 cm^{-1} peak belongs to H_2SO_4 [29]. However, we see no peak around ~858 cm^{-1} even in the fluid entrapped at the most oxidising conditions of NNO+7.4.

Detailed peak fitting of spectra in Figure S1 reveals a systematic increase of the A_{1137}/A_{1144} ratio with increasing temperature (Figure S12). The splitting of the ~1144 cm^{-1} peak may occur due to clustering or complexing of SO_2 molecules[30–32].

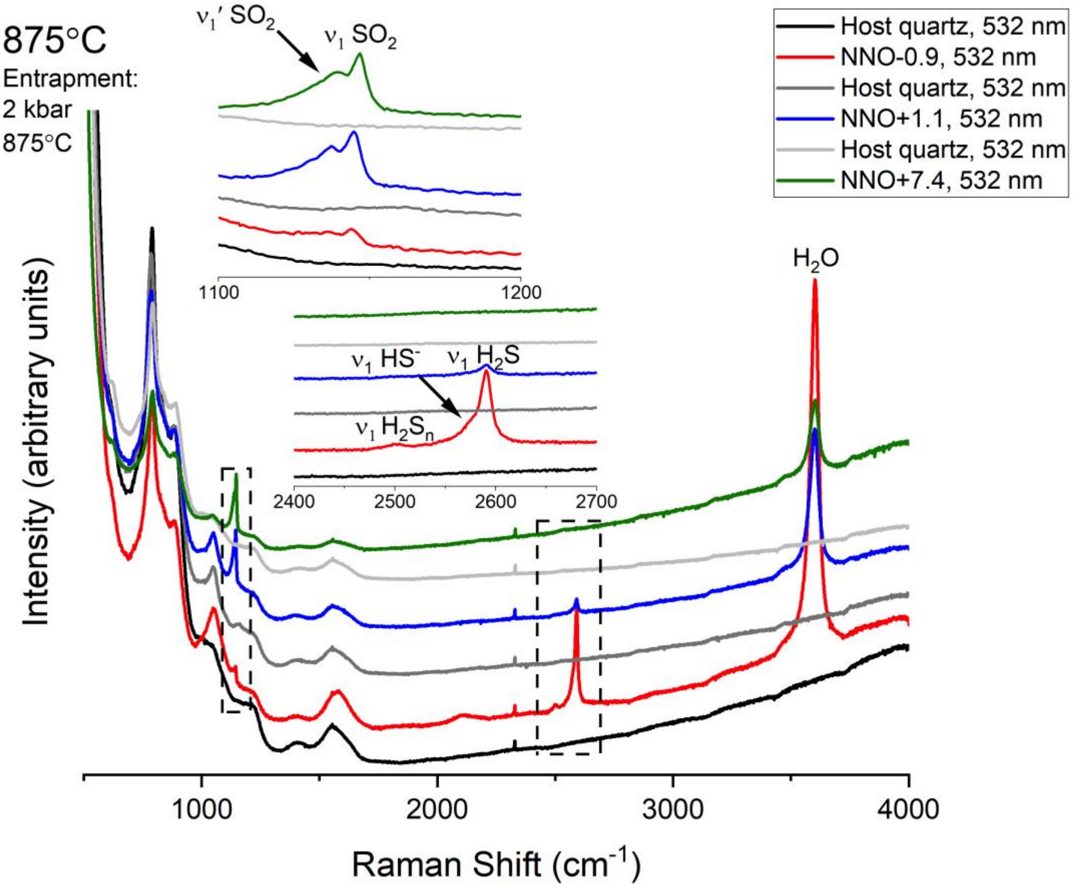

Figure S11. Raw 532 nm Raman spectra of the supercritical fluid phase collected at 875°C and contrasting fO_2 conditions. Note that the ratio of the areas of the ~1137 cm^{-1} and ~1144 cm^{-1} peak remains constant regardless of fO_2.

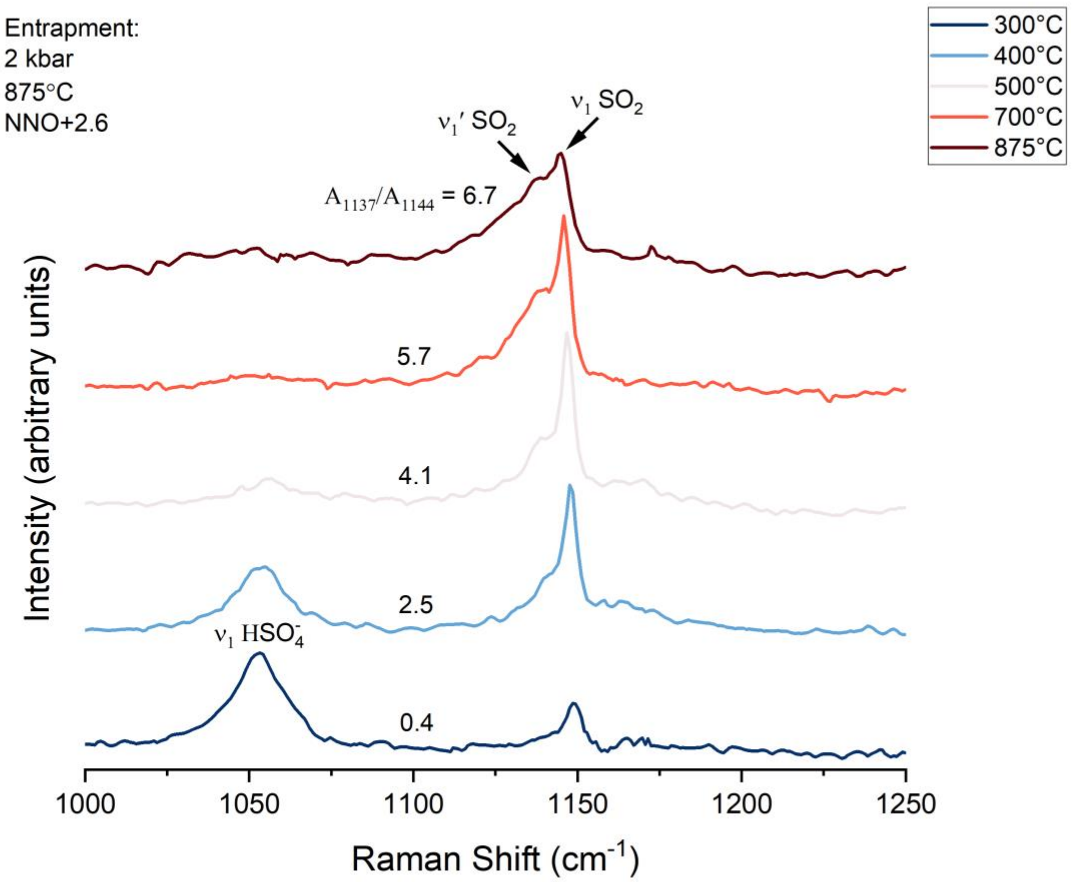

Figure S12. Evolution of the HSO_4^- and SO_2 peaks and the A_{1137}/A_{1144} ratio in the liquid (300-400°C) and supercritical fluid (500-875°C) during heating. Note the decrease of HSO_4^- peak area and the increase of SO_2 peak areas as sulphur comproportionation progresses with increasing temperature. Also note the increase of the A_{1137}/A_{1144} ratio with increasing temperature derived from peak fitting. These spectra are the background corrected versions of raw spectra shown in Figure S1.

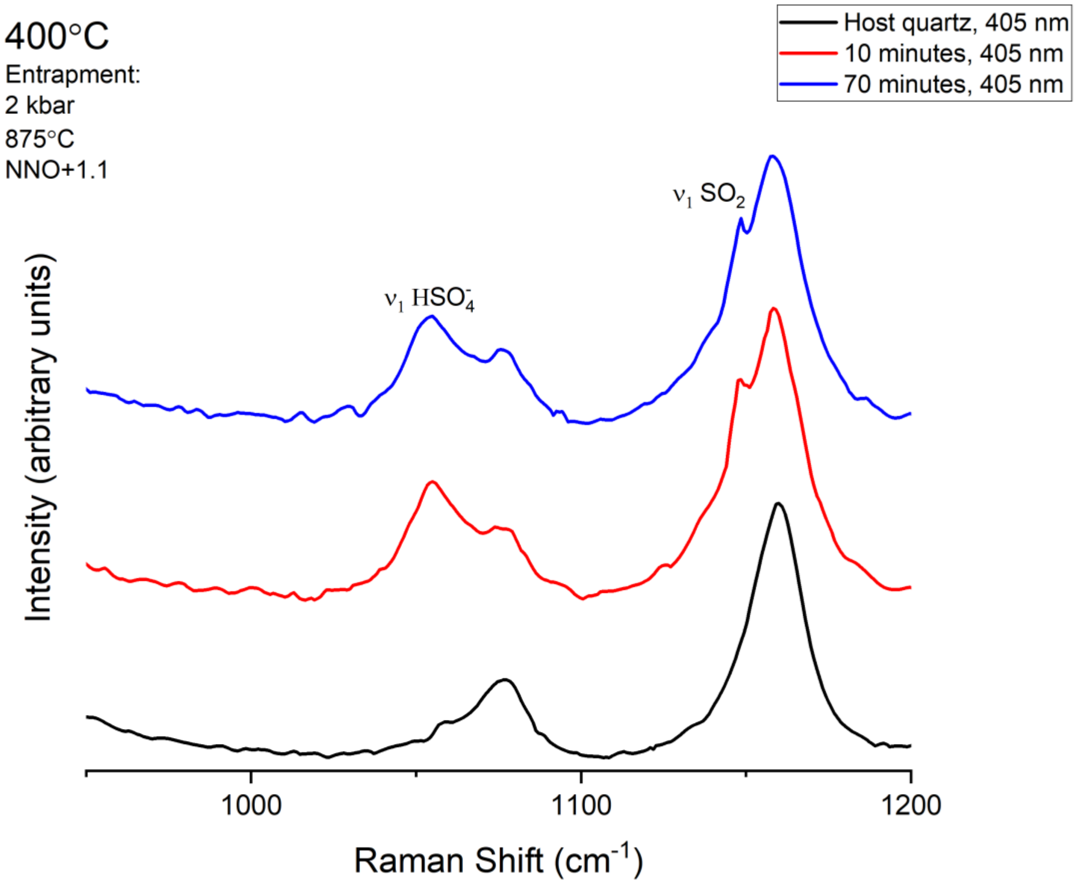

Figure S13. Raw Raman spectra of the liquid phase collected at 400°C after 10 and 70 minutes from reaching this temperature. The ratios of bands of sulphur species remained the same indicating the attainment of thermal and chemical equilibrium within 10 minutes at a moderate temperature (reaction rates increase with increasing temperature). Spectra were vertically offset for better readability.

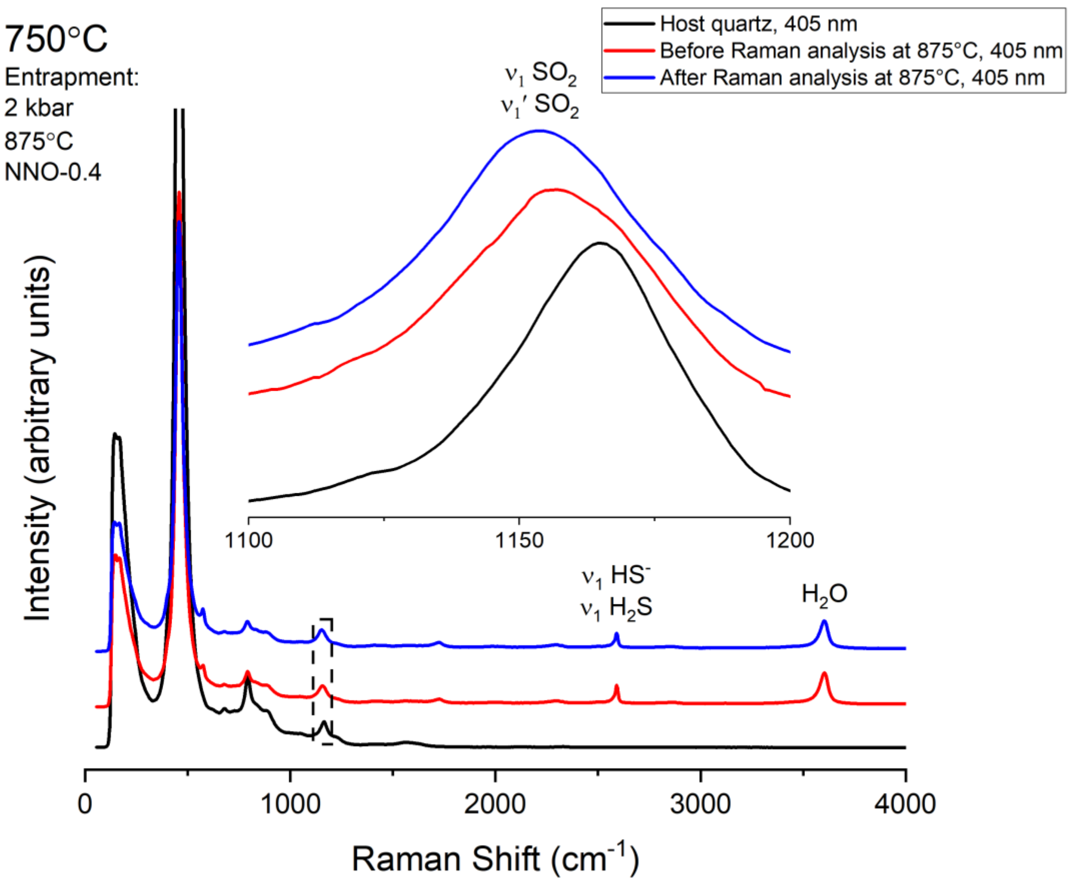

Figure S14. Raw Raman spectra of the supercritical fluid phase collected at 750°C before and after heating and keeping the SFI at 875°C. The oxidation of minor sulphide to SO_2 is indicated by changes in the shape of overlapping quartz+SO_2 peaks. Peak fitting revealed that 0.4% of the total sulphur content of the SFI gets oxidised from sulphide to SO_2 during the heating and analysis of SFI at 875°C. Spectra were vertically offset for better readability.

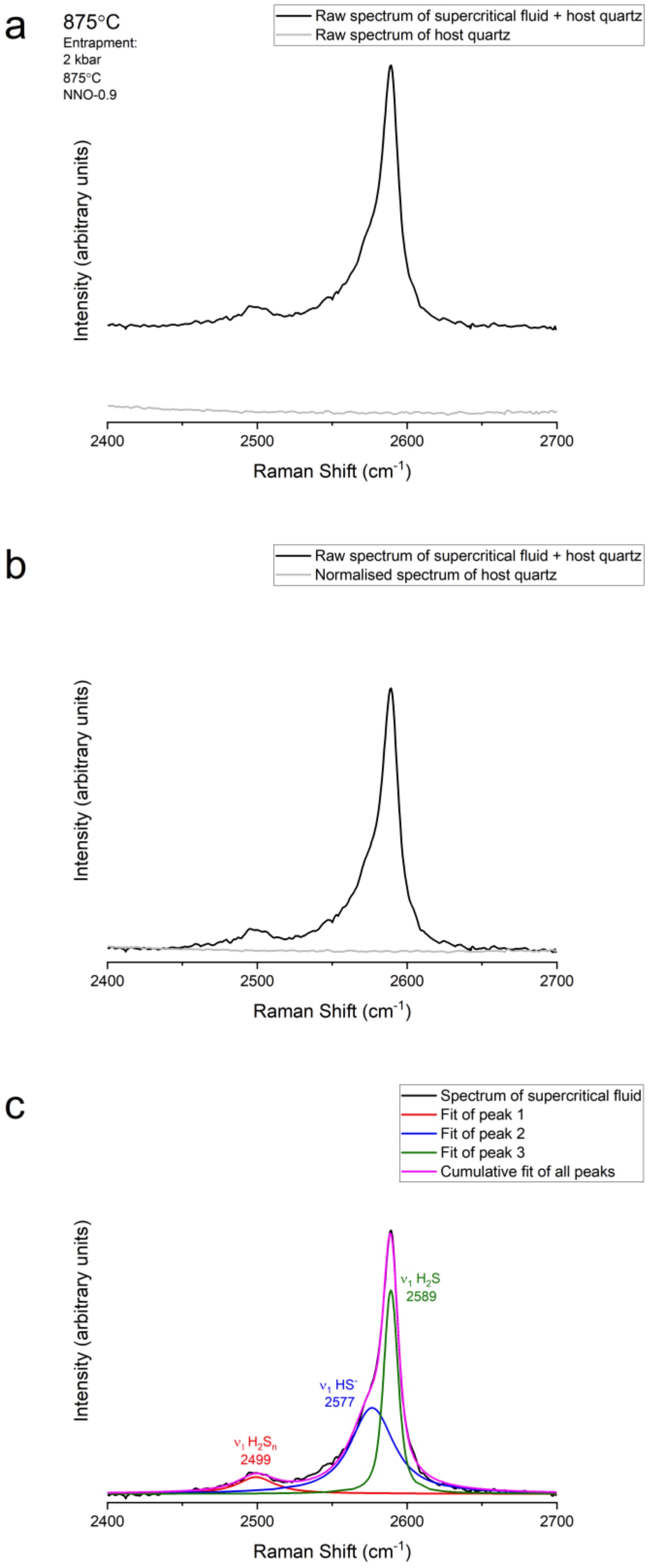
a
875°C
Entrapment:
2 kbar
875°C
NNO-0.9
Raw spectrum of supercritical fluid + host quartz
Raw spectrum of host quartz
Intensity (arbitrary units)
2400
2500
2600
2700
Raman Shift (cm-1)
b
Raw spectrum of supercritical fluid + host quartz
Normalised spectrum of host quartz
Intensity (arbitrary units)
2400
2500
2600
2700
Raman Shift (cm-1)
c
Spectrum of supercritical fluid
Fit of peak 1
Fit of peak 2
Fit of peak 3
Cumulative fit of all peaks
ν1 H2S
2589
ν1 HS-
2577
ν1 H2Sn
2499
Intensity (arbitrary units)
2400
2500
2600
2700
Raman Shift (cm-1)

Figure S15. Correction and peak fitting of Raman spectra: a) The 2400-2700 cm^{-1} spectral region of raw 405 nm Raman spectra of the supercritical fluid phase + host quartz and host quartz collected at the same focal depth. Note that due to the depth of SFI in its host quartz, it is inevitable that part of the excited volume will lie in the host. Given that crystalline quartz is a much better Raman scatterer than the supercritical fluid, Raman spectra of the latter will contain strong quartz bands. b) Intensity of host quartz spectrum normalised to the spectrum of the supercritical fluid phase + host quartz, so the characteristic quartz bands have the same intensity in the two spectra. This is accomplished by iteratively adding a constant and multiplying the spectrum of host quartz in LabSpec until there is a match in intensity of quartz bands. c) The residual Raman spectrum of supercritical fluid acquired by the subtraction of host quartz from that of the supercritical fluid phase + host quartz. This spectrum only contains bands belonging to the supercritical fluid phase and can be peak fitted.

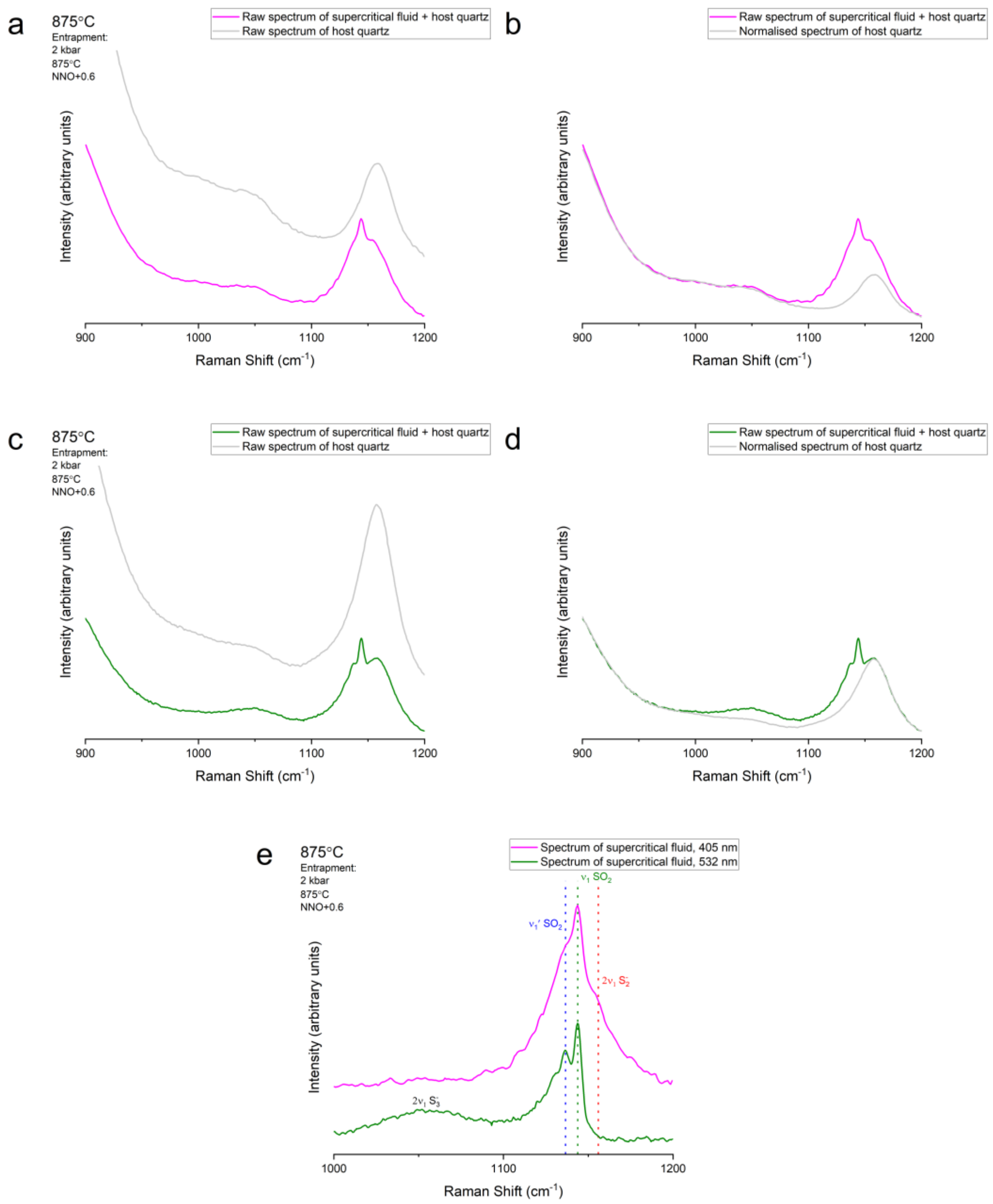

Figure S16. Comparison of Raman spectra of the supercritical fluid phase collected with different wavelengths in the 900-1200 cm^{-1} spectral region. Spectra collected with both 405 nm (a-b) and 532 nm (c-d) excitation were corrected as outlined in Figure S15. In e) the residual Raman spectra of supercritical fluid are visible. The 405 nm spectrum exhibits the $2\nu_1(S_2^-)$ overtone peak and the 532 nm spectrum the $2\nu_1(S_3^-)$ overtone peak. The overlap of three peaks – the $\nu_1'(SO_2)$ peak at ~1137 cm^{-1}, the $\nu_1(SO_2)$ peak at ~1144 cm^{-1}, and the $2\nu_1(S_2^-)$ overtone

peak at ~1158 cm^{-1} – makes peak fitting of 405 nm spectra challenging. Therefore, 532 nm spectra, void of S_2^- spectral features were used to fit the ν_1'(SO_2) and ν_1(SO_2) peaks.

Note that the ν_1(SO_2) and ν_1'(SO_2) peaks corresponding to the symmetric S=O stretching mode of SO_2 display satellite peaks arising from $^{34}SO_2$ at approximately 20 cm^{-1} lower wavenumbers[33]. The area ratio of $\nu_1(^{34}SO_2)/\nu_1(SO_2)$ is ~0.044 [28] and has been considered during quantification.

Raman spectral features of the host α-quartz and its high-temperature polymorphs

The Raman spectrum of the host mineral shows almost all peaks characteristic to α-quartz at 25°C (Table S6, Figure S17). Upon heating, most peaks shift toward lower frequencies and their intensities change, as noted previously[34]. Around 573°C, α-quartz undergoes a transition to β-quartz[34]. Around 870°C, β-quartz should undergo a phase transition to tridymite, however, as indicated by the Raman bands of host, this transition does not occur, consistent with a previous study that failed to observe tridymite even at 1000°C [35]. To distinguish between the peaks of quartz and those of SFI phases and to accurately quantify the latter, Raman spectra of the host phase were collected at each temperature step of each experiment in the horizontal plane of the given inclusion, ~10 µm away from its outer walls.

Fluorescence

In Raman spectra of host quartz collected with the 532 nm excitation, a prominent fluorescence background appears upon heating and reaches a maximum intensity around 300°C. Upon further heating, the background drops to near-zero, before it starts to grow again above 700°C due to thermal incandescence (Extended Data Figure 2). The fluorescence also affects spectra of SFI phases (Figure 3b).

Vibrational mode	Vibrational frequency (cm^{-1})	
	Room temperature<br>Shapiro et al. (1967)	25 °C<br>(this work)
A_1	147	143
A_1	207	206
A_1	355	355
A_1	466	464
A_1	1081	1082
E	128	128
E	264	264
E	394	392
E	403	402
E	452	
E	508	510
E	698	696
E	798	
E	811	808
E	1067	
E	1161	1160
E	1233	1232

Table S6. Vibrational frequencies of the host α-quartz. For the corresponding spectrum see Figure S17.

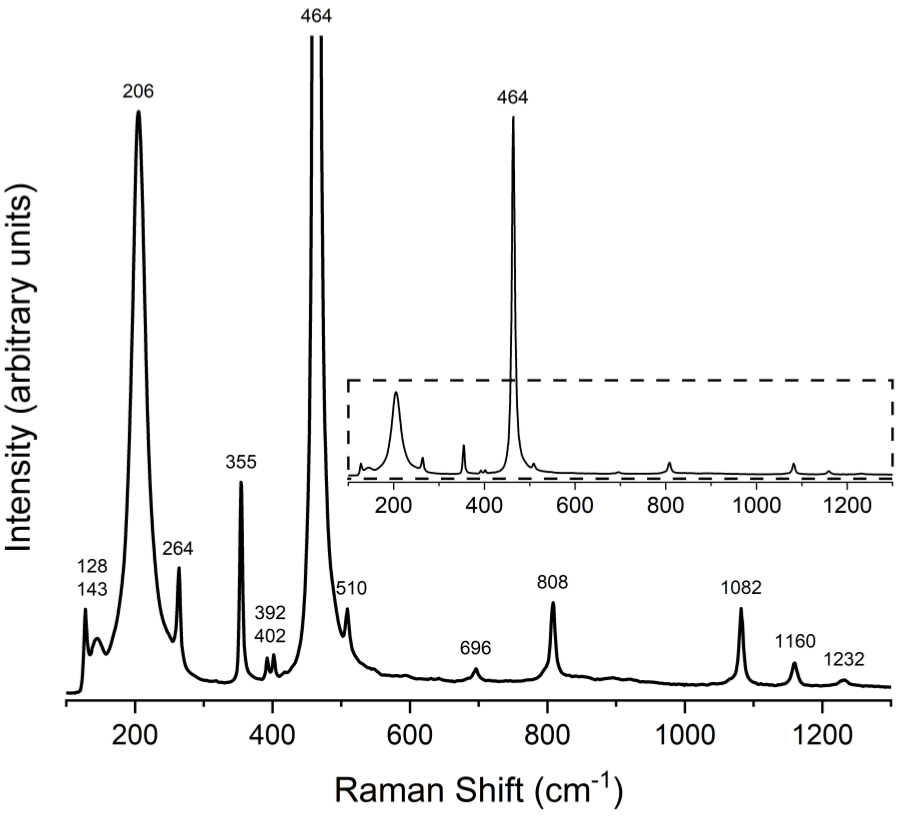

Figure S17. Raw 405 nm Raman spectrum of the α-quartz used for SFI entrapment collected at 25°C.

Raman spectral features of H_2 gas

H_2 gas was detected in room temperature Raman spectra of SFI entrapped between NNO-0.9 and NNO+0.1 (Table S7, Figure 1a). While peak positions of the S0 branch show perfect agreement with previous experimental data[36], peaks of O_1 and Q_1 branches are systematically shifted to higher frequencies by ~3 cm^{-1} (Table S7).

Vibrational mode<br>Veirs and Rosenblatt (1987)		Vibrational frequency (cm^{-1})	
Branch	J	H_2 gas<br>Veirs and Rosenblatt (1987)	Fluid inclusion<br>(this work)
S_0 branch	0	354	
	1	587	587
	2	814	814
	3	1035	1035
	4	1246	1247
	5	1447	1448
O_1 branch	2	3807	3810
	3	3568	3571
	4	3329	
	5	3091	
Q_1 branch	0	4161	4164
	1	4155	4158
	2	4143	4146
	3	4126	4129
	4	4103	4105
	5	4074	4076
	6	4039	

Table S7. Vibrational frequencies of hydrogen gas.

Raman spectral features of sulphur-bearing species

A wide range of sulphur-bearing species has been identified in our SFI based on their well-documented Raman features (Table S8, Figure 1). In the following section, Raman spectral features characteristic of different sulphur-bearing species are discussed.

Native sulphur S_8. At 25°C, pale yellow sulphur daughter crystals are optically visible in SFI grown at all experimental fO_2 conditions (Extended Data Figure 4). When hit with the laser beam, these can be readily identified based on their strong 462 and 220 cm^{-1} peaks[37].

S_n^{2-} polysulphides. The stretching modes of unbranched dianions S_n^{2-} occur only in the region 390–510 cm^{-1} [25]. We have not observed any Raman peaks corresponding to S_n^{2-} polysulfides.

Sulphate SO_4^{2-}. The sulphate ion was identified based on its highest intensity $\nu_1(SO_4^{2-}$) peak at 981 cm^{-1} [38].

Hydrogen sulphate or bisulphate HSO_4^{-}. The bisulphate ion was identified based on its highest intensity peak at 1050 cm^{-1} [39].

Sulphuric acid *H_2SO_4*. Sulphuric acid was not identified in this study. At high temperatures it shows peaks centred at 858 and 1186 cm^{-1} [29].

Sulphur dioxide SO_2. Sulphur dioxide was identified based on its highest intensity $\nu_1(SO_2)$ peak at 1144 cm^{-1} reported above 250°C [23]. Upon heating this peak splits into two – see "Assignment of the ~1137 cm^{-1} peak" section of Supplementary Material.

Sulphur trioxide SO_3. Sulphur trioxide can be identified based on its highest intensity $\nu_1(SO_3)$ peak at 1069 cm^{-1} [40]. We have not observed any Raman peaks corresponding to sulphur trioxide.

Hydrogen sulphide H_2S. Hydrogen sulphide was identified based on its most intense $\nu_1(H_2S)$ peak situated at 2590 and 2610 cm^{-1} in spectra of the liquid and vapour phase,

respectively[39]. Upon homogenization into a supercritical fluid, these peaks merge into one with a frequency of 2593 cm^{-1}. Upon further heating (and pressure increase), the position of this peak shifts gradually to 2589 cm^{-1} at 875°C.

Hydrosulphide or bisulphide HS^-. Bisulphide was identified based its most intense peak at 2570 cm^{-1} [41].

H_2S_n (n>1) polysulphanes. These were identified based on their peaks around 2500 cm^{-1} [42,43].

S_2^- radical. The $\nu_1(S_2^-)$ band at ~582 cm^{-1} corresponds to the symmetric S–S stretching mode[44,45]. Given that the 405 nm excitation line lies inside the absorbance band of the S_2^- radical (Extended Data Figure 2), spectra collected with the 405 nm excitation show a strong enhancement of the intensity of the $\nu_1(S_2^-)$ band and its $n\nu_1$ progression. At conditions at which the strongest S_2^- Raman signal was measured (with 405 nm excitation, at 500°C, and in the fluid inclusion entrapped at 2 kbar, 875°C, and NNO+0.1), even the $6\nu_1$ overtone is visible (Figure 3a).

S_3^- radical. The S_3^- radical has three Raman bands: the $\nu_1(S_3^-)$ band at ~546 cm^{-1}, corresponding to the symmetric S–S stretching mode[44–46], the $\nu_2(S_3^-)$ band at ~258 cm^{-1}, corresponding to the symmetric S–S bending mode[45,46], and the $\nu_3(S_3^-)$ band at ~582 cm^{-1}, corresponding to the anti-symmetric S–S stretching mode[47]. Given that the 532 nm excitation line lies inside the absorbance band of the S_3^- radical (Extended Data Figure 2), spectra collected with the 532 nm excitation show a strong enhancement of the intensity of its bands and its $n\nu_1$ progression. At conditions at which the strongest S_3^- Raman signal was measured (with 532 nm excitation, at 300°C, and in the fluid inclusion entrapped at 2 kbar, 875°C, and NNO-0.4), even the $3\nu_1$ overtone is visible (Figure 3b).

Vibrational mode	Vibrational frequency (cm^{-1})			
	**S**			
Trofimov et al. (2009)	Solid sulphur<br>Ward (1968)	Solid sulphur<br>Trofimov et al. (2009)	Fluid inclusion<br>Giuliani et al. (2003)	Fluid inclusion<br>Jacquemet et al. (2014)
τ	84	85		
δ	151	152		153
δ	218	218	220	221
δ	248	243		
ν	437	434		
ν	474	470	462	469

	**SO_4^{2-}(aq)**						
Hester and Plane (1964)<br>Choi and Lockwood (1989)	Na_2SO_4 solution<br>Hester and Plane (1964)	Na_2SO_4 solid at 295 K<br>Choi and Lockwood (1989)	$(NH_4)_2SO_4$ solution<br>Rudolph (1996)	Li_2SO_4 solution<br>Myhre et al. (2003)	Fluid inclusion<br>Rosasco and Roedder (1979)	Fluid inclusion<br>Dubessy et al. (1992)	Fluid inclusion at 25°C (this work)
ν_1 S–O symmetric stretching	980	977	981	982	981	980	981
ν_2 S–O bending	450	451	452	450			
ν_3 S–O antisymmetric stretching	1105	1131	1110	1122			
ν_4 S–O bending	625	647	617	610			

	**HSO_4^-(aq)**					
	H_2SO_4 solution<br>Savoie and Giguère (1964)	50 mol% NH_4HSO_4 solution at 93°C<br>Irish and Chen (1970)	NH_4HSO_4 solution<br>Rudolph (1996)	NH_4HSO_4 solution<br>Myhre et al. (2003)	Fluid inclusion<br>Dubessy et al. (1992)	Fluid inclusion at 25°C<br>(this work)
ν_1 S–O symmetric stretching	1047	1040	1052	1047	1050	1052
ν_2 S–OH stretching	890	872	898	885	890	
ν_3 S–O asymmetric stretching		1204		1192		
ν_4 S–O symmetric bending &<br>ν_5 S–O asymmetric bending		592	588	593	590	
ν_6 S–OH symmetric bending	409	424	422	425		

	**SO_2(g)**		
Gerding and Nijveld (1937)	Gerding and Nijveld (1937)	In Ar matrix<br>Maillard et al. (1975)	Fluid inclusion at 875°C<br>(this work)
ν_1 S–O symmetric stretching	1151	1147	1137, 1144
ν_2 S–O bending	525	517	
ν_3 S–O antisymmetric stretching	1336	1351	

	**SO_3(g)**
Gerding et al. (1936)	Gerding et al. (1936)
ν_1 S–O symmetric stretching	1069

	**H_2S(aq)**				
	H_2S(l)<br>Murphy and Vance (1938)	Water at 20°C<br>Dubessy et al. (1992)	Fluid inclusion at 20°C<br>Dubessy et al. (1992)	Fluid inclusion at 25°C<br>(this work)	Fluid inclusion at 875°C<br>(this work)
ν_1 S–H symmetric stretching	2574	2590	2580	2591	2589

	**H_2S(g)**		
	H_2S(g)<br>Murphy and Vance (1938)	Fluid inclusion at 20°C<br>Dubessy et al. (1992)	Fluid inclusion at 25°C<br>(this work)
ν_1 S–H symmetric stretching	2611	2610	2613

	**HS^-**		
	$NaOH+H_2S+NaClO_4$ solution<br>Meyer et al. (1983)	Fluid inclusion at 25°C<br>(this work)	Fluid inclusion at 875°C<br>(this work)
ν_1 S–H symmetric stretching	2570	2571	2577

	**H_2S_n(n>1)**		
	von Fehér et al. (1956)	Liquid S phase of the S–H_2S–CH_4–H_2O system at 120–250°C	Fluid inclusion at 875°C<br>(this work)

		Yu et al. (2021)	
ν_1 S–H symmetric stretching	~2500	2500	2499

S_2^-

Holzer et al. (1969) Clark and Franks (1975) Farsang et al. (2023)	Ultramarine blue Clark and Franks (1975)	Lazurite, Russia Farsang et al. (2023)	Synthetic ultramarine blue Farsang et al. (2023)	Fluid inclusion at 500°C (this work)
ν_1 symmetric S–S stretching	587	582	583	576
$2\nu_1$ overtone	1168	1163	1164	1158
$3\nu_1$ overtone	1747	1740	1736	1729
$4\nu_1$ overtone	2320	2314	2310	2301
$5\nu_1$ overtone		2882	2879	2869
$6\nu_1$ overtone		3448	3445	3443
$7\nu_1$ overtone			3967	
$8\nu_1$ overtone			4516	

S_3^-

Holzer et al. (1969) Chivers and Drummond (1972) Clark and Franks (1975) Farsang et al. (2023)	Ultramarine blue Clark and Franks (1975)	Lazurite, Russia Farsang et al. (2023)	Synthetic ultramarine blue Farsang et al. (2023)	Alkali Polysulfide solution in Hexamethyl-phosphoramide Chivers and Drummond (1972)	Aqueous solutions at different P/T Pokrovski and Dubrovinsky (2011)	Fluid inclusion at 300°C (this work)
ν_1 symmetric S–S stretching	549	546	545	533	534-544	531
$2\nu_1$ overtone	1096	1093	1098	1067	1065-1101	1064
$3\nu_1$ overtone	1641	1643	1645	1597	1597-1620	1605
$4\nu_1$ overtone	2187	2187	2181	2126	2134-2152	
$5\nu_1$ overtone	2730	2731	2728			
$6\nu_1$ overtone	3270	3264	3266			
$7\nu_1$ overtone		3795	3797			
$8\nu_1$ overtone		4317	4311			
ν_2 symmetric S–S bending		258	254	232	236-248, 292-295	
$\nu_1 + \nu_2$ overtone	807	805	801		772-780, 818-827	
$2\nu_1 + \nu_2$ overtone	1355	1355	1354			
$3\nu_1 + \nu_2$ overtone	1899	1895	1895		1860	
$4\nu_1 + \nu_2$ overtone		2448				
$5\nu_1 + \nu_2$ overtone		2986				
$6\nu_1 + \nu_2$ overtone		3512				
$7\nu_1 + \nu_2$ overtone		4049				
ν_3 antisymmetric S–S stretching		585				

Table S8. Vibrational frequencies of sulphur-bearing species.

References

1. Pokrovski, G. S. & Dubrovinsky, L. S. The S3- ion is stable in geological fluids at elevated temperatures and pressures. *Science (1979)* **331**, 1052–1054 (2011).

2. Jacquemet, N., Guillaume, D., Zwick, A. & Pokrovski, G. S. In situ Raman spectroscopy identification of the S3- ion in S-rich hydrothermal fluids from synthetic fluid inclusions. *American Mineralogist* **99**, 1109–1118 (2014).

3. Pokrovski, G. S. & Dubessy, J. Stability and abundance of the trisulfur radical ion S3- in hydrothermal fluids. *Earth Planet Sci Lett* **411**, 298–309 (2015).

4. Colin, A. *et al.* In situ determination of sulfur speciation and partitioning in aqueous fluid-silicate melt systems. *Geochem Perspect Lett* **14**, 31–35 (2020).

5. Helgeson, H. C., Delany, J. M., Nesbitt, H. W. & Bird, D. K. Summary and critique of the thermodynamic properties of rock-forming minerals. *Am J Sci* **278-A**, 1–229 (1978).

6. Chase, M. W. *NIST-JANAF Thermochemical Tables*. (American Institute of Physics, New York, 1998).

7. Johnson, J. W., Oelkers, E. H. & Helgeson, H. C. SUPCRT92: A software package for calculating the standard molal thermodynamic properties of minerals, gases, aqueous species, and reactions from 1 to 5000 bar and 0 to 1000°C. *Comput Geosci* **18**, 899–947 (1992).

8. Shock, E. L., Sassani, D. C., Willis, M. & Sverjensky, D. A. Inorganic species in geologic fluids: Correlations among standard molal thermodynamic properties of aqueous ions and hydroxide complexes. *Geochim Cosmochim Acta* **61**, 907–950 (1997).

9. Tagirov, B. R., Zotov, A. V. & Akinfiev, N. N. Experimental study of dissociation of HCl from 350 to 500°C and from 500 to 2500 bars: Thermodynamic properties of HCl°(aq). *Geochim Cosmochim Acta* **61**, 4267–4280 (1997).

10. Sverjensky, D. A., Shock, E. L. & Helgeson, H. C. Prediction of the thermodynamic properties of aqueous metal complexes to 1000°C and 5 kb. *Geochim Cosmochim Acta* **61**, 1359–1412 (1997).

11. Sullivan, N. A. *et al.* The solubility of gold and palladium in magmatic brines: Implications for PGE enrichment in mafic-ultramafic and porphyry environments. *Geochim Cosmochim Acta* **316**, 230–252 (2022).

12. Akinfiev, N. N. & Diamond, L. W. Thermodynamic description of aqueous nonelectrolytes at infinite dilution over a wide range of state parameters. *Geochim Cosmochim Acta* **67**, 613–629 (2003).

13. Shock, E. L. & Helgeson, H. C. Calculation of the thermodynamic and transport properties of aqueous species at high pressures and temperatures: Correlation algorithms for ionic species and equation of state predictions to 5 kb and 1000°C. *Geochim Cosmochim Acta* **52**, 2009–2036 (1988).

14. Robie, R. A., Hemingway, B. S. & Fisher, J. R. *Thermodynamic Properties of Minerals and Related Substances at 298.15 K and 1 Bar (105 Pascals) Pressure and at Higher Temperatures*. (US Government Printing Office, Washington, 1995).

15. Akinfiev, N. N. & Zotov, A. V. Thermodynamic Description of Chloride, Hydrosulfide, and Hydroxo Complexes of Ag(I), Cu(I), and Au(I) at Temperatures of 25–500°C and Pressures of 1–2000 bar. *Geochemistry International* **39**, 1083–1099 (2001).

16. Williams, T. J., Candela, P. A. & Piccoli, P. M. Hydrogen-alkali exchange between silicate melts and two-phase aqueous mixtures: an experimental investigation. *Contrib Mineral Petrol* 114–126 (1997).

17. Hsu, Y. J., Zajacz, Z., Ulmer, P. & Heinrich, C. A. Chlorine partitioning between granitic melt and H2O-CO2-NaCl fluids in the Earth's upper crust and implications for magmatic-hydrothermal ore genesis. *Geochim Cosmochim Acta* **261**, 171–190 (2019).

18. Heinrich, C. A. The physical and chemical evolution of low-salinity magmatic fluids at the porphyry to epithermal transition: A thermodynamic study. *Miner Depos* **39**, 864–889 (2005).

19. Heinrich, C. A. The Chain of Processes Forming Porphyry Copper Deposits — An Invited Paper*. *Economic Geology* **119**, 741–769 (2024).

20. Zajacz, Z., Candela, P. A., Piccoli, P. M. & Sanchez-Valle, C. The partitioning of sulfur and chlorine between andesite melts and magmatic volatiles and the exchange coefficients of major cations. *Geochim Cosmochim Acta* **89**, 81–101 (2012).

21. Zajacz, Z., Candela, P. A., Piccoli, P. M., Sanchez-Valle, C. & Wälle, M. Solubility and partitioning behavior of Au, Cu, Ag and reduced S in magmas. *Geochim Cosmochim Acta* **112**, 288–304 (2013).

22. Binder, B., Wenzel, T. & Keppler, H. The partitioning of sulfur between multicomponent aqueous fluids and felsic melts. *Contributions to Mineralogy and Petrology* **173**, 18 (2018).

23. Ni, H. & Keppler, H. In-situ Raman spectroscopic study of sulfur speciation in oxidized magmatic-hydrothermal fluids. *American Mineralogist* **97**, 1348–1353 (2012).

24. Drummond. Boiling and mixing of hydrothermal fluids: Chemical effects on mineral precipitation. (The Pennsylvania State University, 1981).

25. Steudel, R. & Chivers, T. The role of polysulfide dianions and radical anions in the chemical, physical and biological sciences, including sulfur-based batteries. *Chem Soc Rev* **48**, 3279–3319 (2019).

26. Reinen, D. & Lindner, G. G. The nature of the chalcogen colour centres in ultramarine-type solids. *Chem Soc Rev* **28**, 75–84 (1999).

27. Binder, B. & Keppler, H. The oxidation state of sulfur in magmatic fluids. *Earth Planet Sci Lett* **301**, 190–198 (2011).

28. Schmidt, C. & Seward, T. M. Raman spectroscopic quantification of sulfur species in aqueous fluids: Ratios of relative molar scattering factors of Raman bands of H2S, HS−,SO2, HSO4−, SO42−, S2O32−, S3− and H2O at ambient conditions and information on changes with pressure and temper. *Chem Geol* **467**, 64–75 (2017).

29. Walrafen, G. E., Yang, W. & Chu, Y. C. High-Temperature Raman Investigation of Concentrated Sulfuric Acid Mixtures: Measurement of H-Bond Δ H Values between H3O+ or H5O2+ and HSO4-. *Journal of Physical Chemistry A* **106**, 10162–10173 (2002).

30. Kamoun, M. Evidence for Short Range Orientation Eflects in. *Journal of Raman Spectroscopy* **8**, 225–226 (1979).

31. Swanson, B. I. *et al.* Raman study of SO2 at high pressure: aggregation, phase transformations, and photochemistry. *Chem Phys Lett* **91**, 393–395 (1982).

32. Dhamelincourt, M. C., Wallart, F., Barbier, P., Mairesse, G. & Descroix, P. Raman spectroscopic study of LiAlCl4/SOCl2/SO2 systems. *J Power Sources* **14**, 77–82 (1985).

33. Anderson, A. & Savoie, R. Raman Spectrum of Crystalline and Liquid SO2. *Can J Chem* **43**, 2271–2278 (1965).

34. Shapiro, S. M., O'Shea, D. C. & Cummins, H. Z. Raman scattering study of the alpha-beta phase transition in quartz. *Phys Rev Lett* **19**, 361–364 (1967).

35. Holmquist, S. B. Conversion of Quartz to Tridymite. *Journal of the American Ceramic Society* **44**, 82–86 (1961).

36. Veirs, D. K. & Rosenblatt, G. M. Raman line positions in molecular hydrogen: H2, HD, HT, D2, DT, and T2. *J Mol Spectrosc* **121**, 401–419 (1987).

37. Giuliani, G. *et al.* CO2-H2S-COS-S8-AlO(OH)-bearing fluid inclusions in ruby from marble-hosted deposits in Luc Yen area, North Vietnam. *Chem Geol* **194**, 167–185 (2003).

38. Rosasco, G. J. & Roedder, E. Application of a new Raman microprobe spectrometer to nondestructive analysis of sulfate and other ions in individual phases in fluid inclusions in minerals. *Geochim Cosmochim Acta* **43**, 1907–1915 (1979).

39. Dubessy, J., Boiron, M.-C., Moissette, A., Monnin, C. & Sretenskaya, N. Determinations of water, hydrates and pH in fluid inclusions by micro-Raman spectrometry. *European Journal of Mineralogy* **4**, 885–894 (1992).

40. Gerding, H., Nijveld, W. J. & Muller, G. J. Raman Effect of Gaseous and Liquid Sulphur Trioxide and of Mixtures of the Trioxide with the Dioxide. *Nature* **137**, 1033 (1936).

41. Meyer, B., Ward, K., Koshlap, K. & Peter, L. Second Dissociation Constant of Hydrogen Sulfide. *Inorg Chem* **22**, 2345–2346 (1983).

42. von Fehér, F., Laue, W. & Winkhaus, G. Beiträge zur Chemie des Schwefels. XXX. Über die Darstellung der Sulfane H2S2, H2S3, H2S4 und H2S5. *Z Anorg Allg Chem* **288**, 113–240 (1956).

43. Yu, Y. *et al.* Species of sulfur in sour gas reservoir: Insights from in situ Raman spectroscopy of S-H2S-CH4-H2O system and its subsystems from 20 to 250°C. *Geofluids* **2021**, (2021).

44. Holzer, W., Murphy, W. F. & Bernstein, H. J. Raman Spectra of Negative Molecular Ions Doped in Alkali Halide Crystals. *J Mol Spectrosc* **32**, 13–23 (1969).

45. Clark, R. J. H. & Franks, M. L. The resonance Raman spectrum of ultramarine blue. *Chem Phys Lett* **34**, 69–72 (1975).

46. Chivers, T. & Drummond, I. Characterization of the Trisulfur Radical Anion S3- in Blue Solutions of Alkali Polysulfides in Hexamethylphosphoramide. *Inorg Chem* **11**, 2525–2527 (1972).

47. Ledé, B. *et al.* Observation of the ν3 Raman band of S3- inserted into sodalite cages. *Journal of Raman Spectroscopy* **38**, 1461–1468 (2007).

48. Trofimov, B. A., Sinegovskaya, L. M. & Gusarova, N. K. Vibrations of the S-S bond in elemental sulfur and organic polysulfides: A structural guide. *Journal of Sulfur Chemistry* **30**, 518–554 (2009).

49. Ward, A. T. Raman spectroscopy of sulfur, sulfur-selenium, and sulfur-arsenic mixtures. *J Phys Chem* **72**, 4133–4139 (1968).

50. Hester, R. E. & Plane, R. A. A Raman Spectrophotometric Comparison of Interionic Association in Aqueous Solutions of Metal Nitrates, Sulfates, and Perchlorates. *Inorg Chem* **3**, 769–770 (1964).

51. Choi, B.-K. & Lockwood, D. J. Raman spectrum of Na2SO4 (Phase V). *Solid State Commun* **72**, 133–137 (1989).

52. Rudolph, W. Structure and dissociation of the hydrogen sulphate ion in aqueous solution over a broad temperature range: A Raman study. *Zeitschrift für Physikalische Chemie* **194**, 73–95 (1996).

53. Myhre, C. E. L., Christensen, D. H., Nicolaisen, F. M. & Nielsen, C. J. Spectroscopic Study of Aqueous H2SO4 at Different Temperatures and Compositions: Variations in Dissociation and Optical Properties. *Journal of Physical Chemistry A* **107**, 1979–1991 (2003).

54. Savoie, R. & Giguère, P. A. Infrared study of the crystalline monohydrates of nitric, perchloric, and sulfuric acids. *J Chem Phys* **41**, 2698–2705 (1964).

55. Irish, D. E. & Chen, H. Equilibria and Proton Transfer in the Bisulfate-Sulfate System. *J Phys Chem* **74**, 3796–3801 (1970).

56. Gerding, H. & Nijveld, W. J. The Raman spectrum of sulphur dioxide in different states. *Recueil des Travaux Chimiques des Pays-Bas* **56**, 968–982 (1937).

57. Maillard, D., Allavena, M. & Perchard, J. P. Spectres vibrationnels du dioxyde de soufre dans une matrice d'argon, d'azote et de xénon. *Spectrochim Acta A* **31**, 1523–1531 (1975).

58. Murphy, G. M. & Vance, J. E. Raman spectra of hydrogen and deuterium sulfides in the gas, liquid and solid states. *J Chem Phys* **6**, 426–429 (1938).

59. Farsang, S., Caracas, R., Adachi, T., Schnyder, C. & Zajacz, Z. S2– and S3– radicals and the S42– polysulfide ion in lazurite, haüyne and synthetic ultramarine blue revealed by resonance Raman spectroscopy. *American Mineralogist* **108**, 2234–2243 (2023).
